# Supplementary material for: Age is just a number: Examining the preservation of cells and soft tissues in Bothriolepis and other Devonian fish
Source: PLoS One. 2025 Dec 3;20(12):e0335783. doi: 10.1371/journal.pone.0335783 (PMC12674576; doi:10.1371/journal.pone.0335783)
Supplement: S1 File — (DOCX) [file pone.0335783.s001.docx]

SUPPORTING INFORMATION

**Age is just a number: Examining the preservation of cells and soft tissues in Bothriolepis and other Devonian fish**

Christopher L. Rogoff and Paul V. Ullmann*

*Corresponding author. Email: paul.ullmann@und.edu

Supplemental details on demineralization assay results

Despite their histological similarities and burial largely within the same lithology and paleoenvironmental context (sandy siltstones deposited in fluvial overbank settings), the nine fossil bone specimens were found to demineralize into particulate “debris” at vastly different rates, ranging from 4 (Bothriolepidae indet.) to 14 (*Holoptychius*) weeks. The first sample to breakdown sufficiently for imaging was NUFV 1586, followed by *Psammosteus*, *Hyneria*, *Bothriolepis*, and both *Gyracanthus* samples (Table B below). The second fragment of *Megalichthys* took several weeks longer to breakdown than the first *Megalichthys* scale fragment. Among the bone samples, the *Holoptychius* specimen was the most resilient against demineralization, taking a full three months to breakdown sufficiently for imaging.

During SEM imaging, one *Hyneria* ‘osteocyte’ was found to exhibit both rough and smooth surface textures of contrasting elemental compositions which, upon close inspection, were found to overlap one another. Areas of rough surface texture corresponded with enrichment primarily in Fe and O whereas smooth areas showed enrichment primarily in Si and O (Figure D below), indicating it was preserved as a carbon and sodium-rich goethite “shell” that later became permineralized by aluminum-rich quartz. No other ‘osteocytes’ from any of the bone samples exhibited this peculiar style of dual preservation, or evidence of being hollow (such as fractures through the cell body making a window into an internal, dark cavity), so this appears to have been a preservation pathway that was spatially restricted to a minority of cellular lacunae within the *Hyneria* bone sample.

Dark red and roughly-spherical intravascular microstructures ~15–20 µm in diameter were also observed in a few ‘vessel’ fragments recovered from *Hyneria* and *Gyracanthus*, similar to those encountered in many previous bone demineralization studies [1,8,34,54,55]. Several authors have interpreted such intravascular microstructures to potentially represent degraded remains of red blood cells [36,54-56], but they have occasionally also been identified as diagenetic (and thus inorganic) precipitates of pyrite, hematite, or clay minerals [e.g., 57,58]. Thus, in the absence of histochemical data, the identity of these intravascular structures in the Devonian specimens remains uncertain.


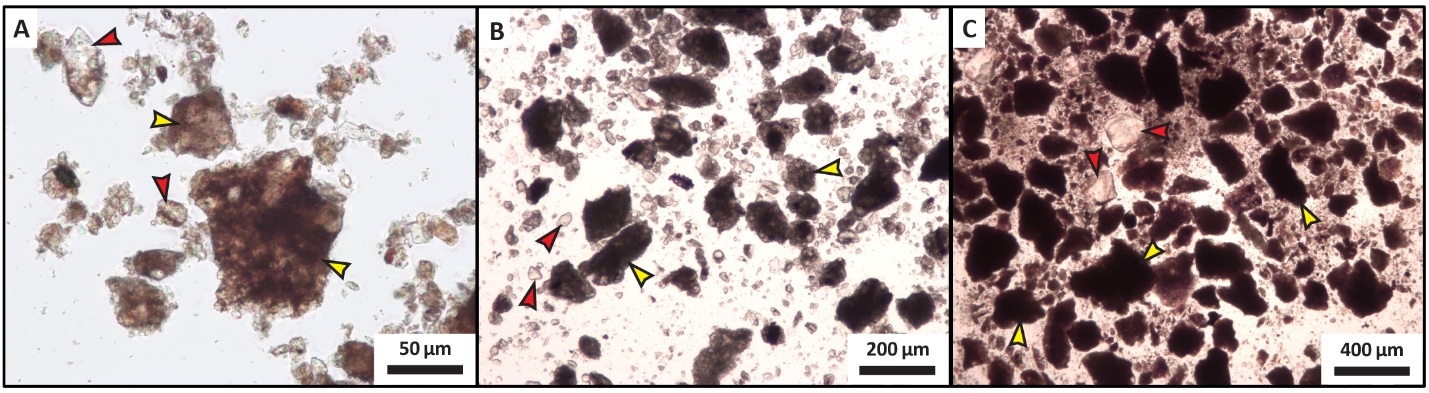


Fig. A. Representative demineralization products recovered from each sediment control. (A) Examples of subangular quartz silt grains and rough, granular fragments of silt and clay-rich mudrock recovered from the Red Hill locality sediment sample. (B) Subangular silt, clay-rich mudrock, and very fine to fine size quartz sand grains recovered from the sediment sample from Canadian locality NV2K11, from which *Psammosteus* sample NUFV 1587 was collected. (C) Subangular to subrounded grains of clay-rich mudrock and fine quartz sand recovered from the sediment sample from Canadian locality NV2K17, which is representative of the sediments from the nearby NV0403 locality [26] where Bothriolepidae indet. sample NUFV 1586 was collected. Red arrows denote example grains of quartz sand and silt, and yellow arrows denote example clay-rich grains of mudrock. Scale bars as indicated.


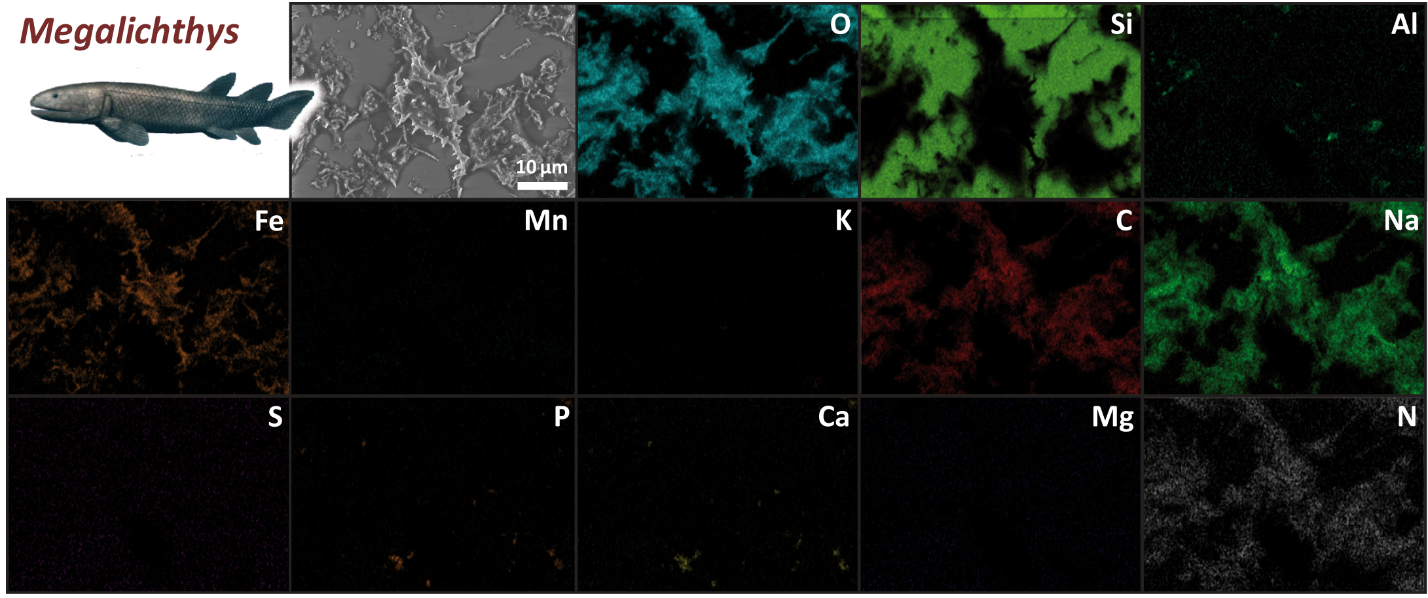


Fig. B. Elemental maps of an ‘osteocyte’ recovered from demineralization products of *Megalichthys*. As revealed in the elemental maps, the ‘osteocyte’ (at center in each panel) is primarily composed of O, Fe, C, Na, and N. Scale bar as indicated in the SEM micrograph. *Megalichthys* reconstruction drawing by Nobu Tamura (published under a CC-BY-SA 3.0 license).


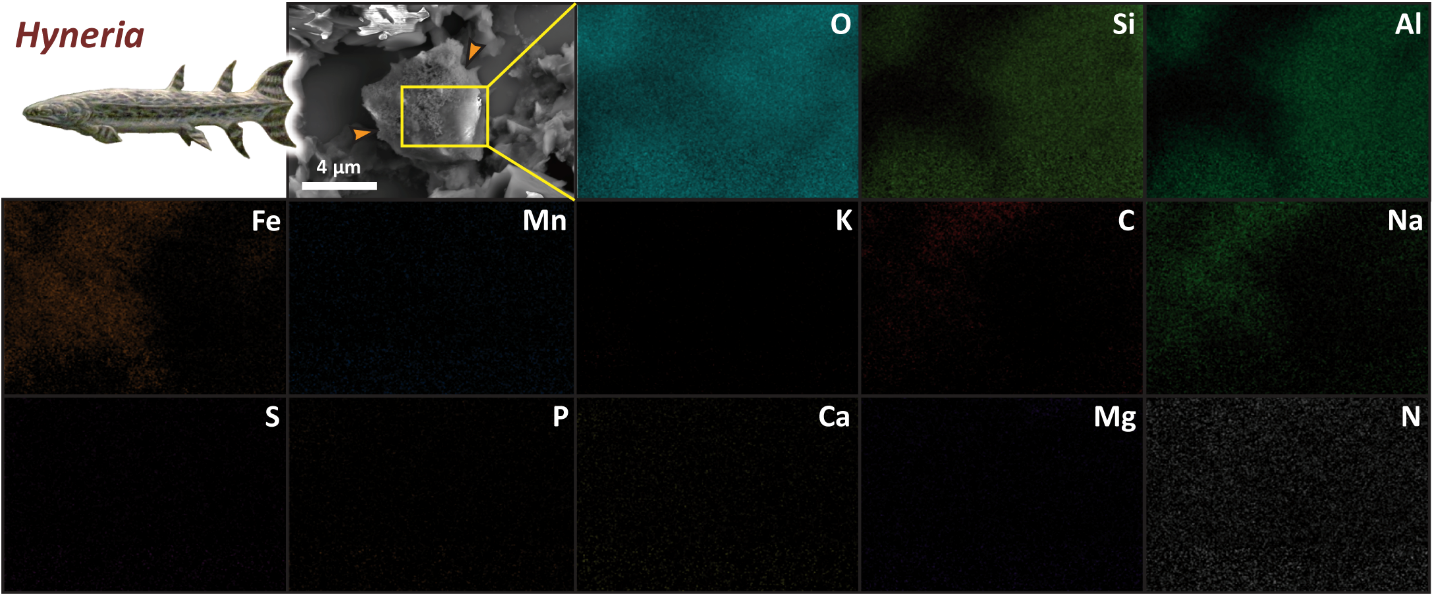


Fig. C. Elemental maps of an ‘osteocyte’ recovered from the demineralization products of *Hyneria* sample ANSP 25034 which exhibits a peculiar, dual mode of preservation. As revealed in the elemental maps, the left portion of the ‘cell body’ of the ‘osteocyte’ exhibits a composition dominated by Fe, O, Na, and C, whereas the right portion is primarily composed of Si, O, and Al. Visual inspection of the SEM micrograph indicates that the iron-rich phase, most likely goethite (to the top and left from the orange arrowheads), forms a thin “shell” over the surface of the underlying silicon-rich phase, most likely quartz (below and to the right of the orange arrowheads), evidencing a two-phase preservation pathway through diagenesis. Scale bar as indicated in the SEM micrograph. *Hyneria* reconstruction drawing by ABelov2014 (published under a CC-BY-SA 3.0 license).


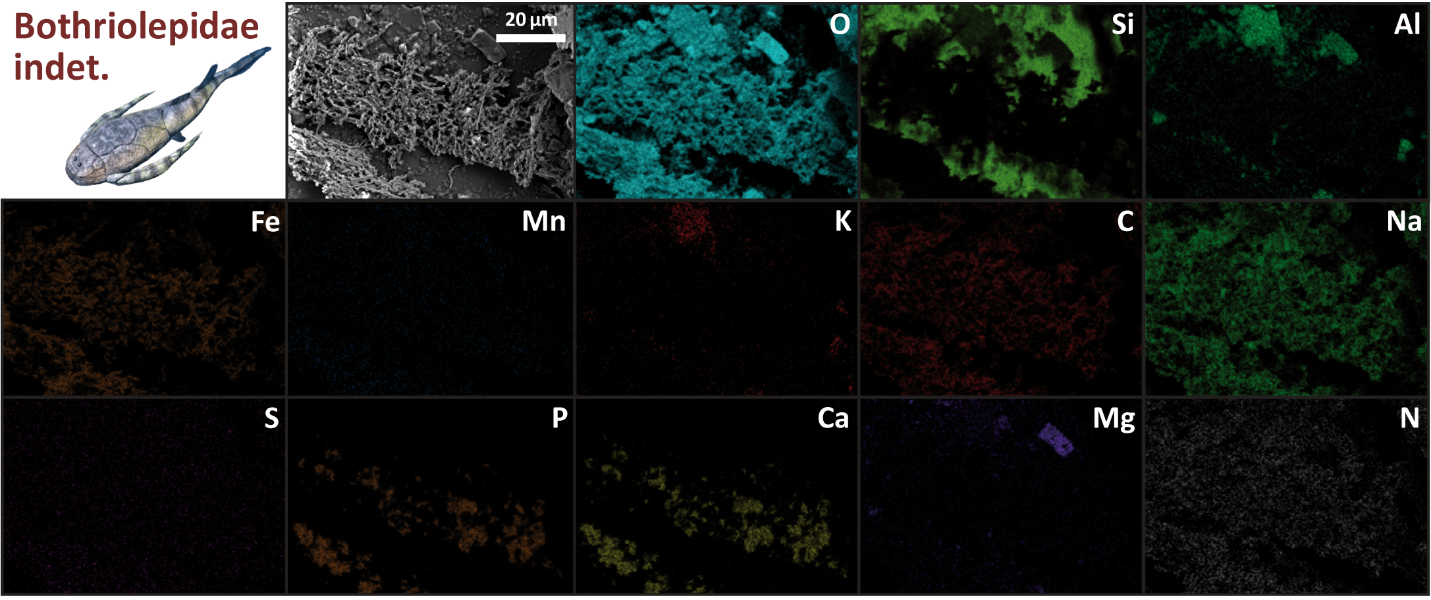


Fig. D. Elemental maps of a fragment of ‘fibrous matrix’ recovered from demineralization products of Bothriolepidae indet. specimen NUFV 1586. As revealed in the elemental maps, the ‘fibrous matrix’ fragment, which crosses from upper left to lower right in each panel, is primarily composed of O, Fe, C, Na, and N. P and Ca cooccur in isolated “patches” within the ‘matrix’, indicative of the presence of remnant, undemineralized fossil bone apatite. Scale bar as indicated in the SEM micrograph. *Bothriolepis* reconstruction drawing by Nobu Tamura (published under a CC-BY-SA 3.0 license).

| **Specimen #** | **Major Clade** | **Minor Clade** | **Taxon** | **Skeletal Element** | **Bioapatitic Tissue Type(s)** | **Formation** | **Locality** |
| --- | --- | --- | --- | --- | --- | --- | --- |
| NUFV 1587 | Pteraspidomorphi | Heterostraci | *Psammosteus* sp. | Dermal bone | Orthodentine, aspidin, acellular lamellar bone | Nordstrand Point | Canada, NV2K11 |
| ANSP uncat. | “Placodermi” | Antiarchi | *Bothriolepis* sp. | Anteroventrolateral plate | Cellular bone | Catskill | Powys Curve |
| NUFV 1586 | “Placodermi” | Antiarchi | Bothriolepidae indet. | Anteroventrolateral plate | Cellular bone | Fram | Canada, NV0403 |
| ANSP uncat. | Eugnathostomata | “Acanthodii” | *Gyracanthus sherwoodi* | Pectoral fin spine | Osteodentine, orthodentine, cellular bone | Catskill | Red Hill |
| ANSP uncat. | Eugnathostomata | “Acanthodii” | *Gyracanthus sherwoodi* | Pectoral fin spine | Osteodentine, orthodentine, cellular bone | Catskill | Red Hill |
| ANSP 23543 | Osteichthyes | Porolepiformes | *Holoptychius* sp. | Scale | Cellular bone | Catskill | Tioga Rest Stop |
| ANSP 21165 | Osteichthyes | Megalichthyidae | *Megalichthys mullisoni* | Scale | Cellular bone, orthodentine, enamel | Catskill | Red Hill |
| ANSP 21165 | Osteichthyes | Megalichthyidae | *Megalichthys mullisoni* | Scale | Cellular bone, orthodentine, enamel | Catskill | Red Hill |
| ANSP 25034 | Osteichthyes | Tristichopteridae | *Hyneria lindae* | Scale | Cellular bone | Catskill | Red Hill |
| NV2K11 sediment |  |  |  |  |  | Nordstrand Point | Canada, NV2K11 |
| Red Hill sediment |  |  |  |  |  | Catskill | Red Hill |
| NV2K17 sediment |  |  |  |  |  | Fram | Canada, NV2K17 |

Table A. Detailed summary of the Devonian fossils and associated sediment samples examined in this study. Specimens are listed in approximate evolutionary order according to the first appearance of the major clade to which they belong. Abbreviations: ANSP, Academy of Natural Sciences of Drexel University (formerly “of Philadelphia”); NUFV, Nunavut Fossil Vertebrate Collection at the Canadian Museum of Nature in Ottawa, Ontario, Canada; uncat, uncatalogued.

| **Specimen #** | **Taxon** | **Tissue Source(s)** | **Demin Order** | **‘Osteo-cytes’** | **‘Osteo’ Shape** | **‘Osteo’ Appearance** | **‘Vessels’ (Light)** | **‘Vessels’ (Dark)** | **‘Vessels’ (Total)** | **‘Fibrous Matrix’** | **Notes** |
| --- | --- | --- | --- | --- | --- | --- | --- | --- | --- | --- | --- |
| NUFV 1587 | *Psammosteus* sp. | Primarily orthodentine and aspidin | 2 | N/A | N/A | N/A | Few | Few | Few | Uncommon | Dark colored ‘fibrous matrix’ (light brown) |
| ANSP uncat. | *Bothriolepis* sp. | Cellular bone | 4 | Abundant | Stellate and flattened-oblate | Dark red | Absent | Few | Few | Uncommon | Yielded more ‘matrix’ than all other samples |
| NUFV 1586 | Bothriolepidae indet. | Cellular bone | 1 | Abundant | Stellate and flattened-oblate | Dark red and light brown | Absent | Few | Few | Few | Exquisite preservation of ‘osteocyte filopodia’ |
| ANSP uncat. | *Gyracanthus sherwoodi* | Primarily osteodentine | 6 | Abundant | Stellate and flattened-oblate | Dark red | Uncommon | Frequent | Frequent | Few | Distinct compositional variation among ‘vessels’ |
| ANSP uncat. | *Gyracanthus sherwoodi* | Primarily osteodentine | 7 | Abundant | Stellate and flattened-oblate | Dark red | Uncommon | Frequent | Frequent | Absent | Both hollow and solid/permineralized ‘vessels’ recovered |
| ANSP 23543 | *Holoptychius* sp. | Cellular bone | 10 | Uncommon | Primarily stellate | Dark brown/red | Absent | Abundant | Abundant | Few | ‘Osteocytes’ almost entirely missing ‘filopodia’ |
| ANSP 21165 | *Megalichthys mullisoni* | Primarily cellular bone | 8 | Very abundant | Stellate and flattened-oblate | Dark red | Few | Uncommon | Uncommon | Few | Exquisite preservation of ‘osteocyte filopodia’ |
| ANSP 21165 | *Megalichthys mullisoni* | Primarily cellular bone | 9 | Very abundant | Stellate and flattened-oblate | Dark red | Few | Uncommon | Uncommon | Few | Very few light-colored ‘osteocytes’, mostly dark ‘osteocytes’ |
| ANSP 25034 | *Hyneria lindae* | Cellular bone | 3 | Very abundant | Primarily stellate | Dark and light red | Few | Uncommon | Uncommon | Few | Yielded hundreds of ‘osteocytes’ |
| NV2K11 sediment | N/A | N/A | 12 | Absent | N/A | N/A | Absent | Absent | Absent | Absent | Yielded no organic microstructures |
| Red Hill sediment | N/A | N/A | 11 | Absent | N/A | N/A | Absent | Absent | Absent | Absent | Yielded no organic microstructures |
| NV2K17 sediment | N/A | N/A | 5 | Absent | N/A | N/A | Absent | Absent | Absent | Absent | Yielded no organic microstructures |

Table B. Detailed summary of cellular and soft tissue recovery from the Devonian fossils and associated sediment samples examined in this study. As in Table 1 and Table A above, the fossil specimens are listed in approximate evolutionary order according to the first appearance of the major clade to which they belong, followed by the sediment samples. An additional abundance category compared to those used in Table 1, Very Abundant, was added to denote specimens yielding > 100 of a given type of cellular/soft-tissue microstructure. Abbreviations: ANSP, Academy of Natural Sciences of Drexel University (formerly “of Philadelphia”); Demin, demineralization; NUFV, Nunavut Fossil Vertebrate Collection at the Canadian Museum of Nature in Ottawa, Ontario, Canada; Osteo, ‘osteocyte’; uncat, uncatalogued.

Supplementary References

54. M. H. Schweitzer, J. R. Horner, Intravascular microstructures in trabecular bone tissues of *Tyrannosaurus rex*. *Annales de Paleontologie* **85**, 179–192 (1999).

55. S. Bertazzo, S. C. R. Maidment, C. Kallepitis, S. Fearn, M. M. Stevens, H. Xie, Fibres and cellular structures preserved in 75-million-year-old dinosaur specimens. *Nature Communications* **6**, 7352 (2015).

56. Y.-C. Lee, C.-C. Chiang, P.-Y. Huang, C.-Y. Chung, T. D. Huang, C.-C. Wang, C.-I. Chen, R.-S. Chang, C.-H. Liao, R. R. Reisz, Evidence of preserved collagen in an Early Jurassic sauropodomorph dinosaur revealed by synchrotron FTIR microspectroscopy. *Nature Communications* **8**, 14220 (2017).

57. D. M. Martill, D. M. Unwin, Small spheres in fossil bones: blood corpuscles or diagenetic products? *Palaeontology* **40**, 619–624 (1997).

58. D. E. Korneisel, S. J. Nesbitt, S. Werning, S. Xiao, Putative fossil blood cells reinterpreted as diagenetic structures. *PeerJ* **9**, e12651 (2021).
