# Supplementary material for: Age is just a number: Examining the preservation of cells and soft tissues in Bothriolepis and other Devonian fish
Source: PLoS One. 2025 Dec 3;20(12):e0335783. doi: 10.1371/journal.pone.0335783 (PMC12674576; doi:10.1371/journal.pone.0335783)
Supplement: Attachment — Submitted filename: PONE-D-25-33219.pdf [file pone.0335783.s002.pdf]

PLOS ONE

Age is just a number: Examining the preservation of cells and soft tissues in Bothriolepis and other Devonian fish

--Manuscript Draft--

**Manuscript Number:**	PONE-D-25-33219
**Article Type:**	Research Article
**Full Title:**	Age is just a number: Examining the preservation of cells and soft tissues in Bothriolepis and other Devonian fish
**Short Title:**	Soft tissue preservation in Devonian fossils
**Corresponding Author:**	Paul Ullmann<br>University of North Dakota<br>Grand Forks, ND UNITED STATES OF AMERICA
**Keywords:**	fossil bone; soft tissue preservation; Devonian; Paleozoic; molecular paleontology; demineralization; osteocytes; SEM
**Abstract:**	Recent microscopy and proteomic studies demonstrate fossil bones can preserve remarkable palimpsests of cellular and soft tissue evolution, yet it remains unclear if there are temporal limits to such preservation. To test this, we demineralized nine bone fragments of Bothriolepis and other Late Devonian fish. Removal of apatite from the fossils released numerous microstructures morphologically consistent with vertebrate osteocytes, pieces of blood vessels, and sheets of fibrous bone matrix which were frequently found to exhibit elemental chemistry potentially indicative of partial organic composition. These discoveries extend this style of exceptional preservation to aspidin and dentine and predate all prior reports of similar cellular/soft-tissue preservation in fossil bones by nearly 100 million years, indicating geologic age is not a limiting factor. Thus, this preservation pathway likely began contemporaneously with the evolution of vascularized cellular bone in the early Paleozoic.
**Order of Authors:**	Christopher L Rogoff
	Paul V Ullmann
**Additional Information:**	
**Question**	**Response**
**Financial Disclosure**<br><br>Enter a financial disclosure statement that describes the sources of funding for the work included in this submission. Review the submission guidelines for detailed requirements. View published research articles from *PLOS ONE* for specific examples.<br><br>This statement is required for submission and **will appear in the published article** if the submission is accepted. Please make sure it is accurate.	Yes

Powered by Editorial Manager® and ProduXion Manager® from Aries Systems Corporation

**Funded studies**<br>Enter a statement with the following details:<br>• Initials of the authors who received each award<br>• Grant numbers awarded to each author<br>• The full name of each funder<br>• URL of each funder website<br>• Did the sponsors or funders play any role in the study design, data collection and analysis, decision to publish, or preparation of the manuscript?<br><br>Did you receive funding for this work?	
Please add funding details.<br>as follow-up to "**Financial Disclosure**<br><br>Enter a financial disclosure statement that describes the sources of funding for the work included in this submission. Review the submission guidelines for detailed requirements. View published research articles from *PLOS ONE* for specific examples.<br><br>This statement is required for submission and **will appear in the published article** if the submission is accepted. Please make sure it is accurate.<br><br>**Funded studies**<br>Enter a statement with the following details:<br>• Initials of the authors who received each award<br>• Grant numbers awarded to each author<br>• The full name of each funder<br>• URL of each funder website<br>• Did the sponsors or funders play any role in the study design, data collection and analysis, decision to publish, or preparation of the manuscript?<br><br>Did you receive funding for this work?"	PVU received financial support for this research from Rowan University. The funder did not play any role in the study design, data collection and analysis, decision to publish, or preparation of the manuscript.
Please select the country of your main research funder (please select carefully as in some cases this is used in fee calculation).	UNITED STATES - US

as follow-up to "**Financial Disclosure**<br><br>Enter a financial disclosure statement that describes the sources of funding for the work included in this submission. Review the submission guidelines for detailed requirements. View published research articles from *PLOS ONE* for specific examples.<br><br>This statement is required for submission and **will appear in the published article** if the submission is accepted. Please make sure it is accurate.<br><br>**Funded studies**<br>Enter a statement with the following details:<br>• Initials of the authors who received each award<br>• Grant numbers awarded to each author<br>• The full name of each funder<br>• URL of each funder website<br>• Did the sponsors or funders play any role in the study design, data collection and analysis, decision to publish, or preparation of the manuscript?<br><br>Did you receive funding for this work?"	
**Competing Interests**<br><br>Use the instructions below to enter a competing interest statement for this submission. On behalf of all authors, disclose any competing interests that could be perceived to bias this work—acknowledging all financial support and any other relevant financial or non-financial competing interests.<br><br>This statement is required for submission and **will appear in the published article** if the submission is accepted. Please make sure it is accurate and that any funding sources listed in your Funding Information later in the submission form are also declared in your Financial Disclosure statement.	The authors have declared that no competing interests exist.

Powered by Editorial Manager® and ProduXion Manager® from Aries Systems Corporation

View published research articles from *PLOS ONE* for specific examples.<br><br>**NO authors have competing interests**<br><br>Enter: *The authors have declared that no competing interests exist.*<br><br>**Authors with competing interests**<br><br>Enter competing interest details beginning with this statement:<br><br>*I have read the journal's policy and the authors of this manuscript have the following competing interests: [insert competing interests here]*<br><br>* typeset	
**Ethics Statement**<br><br>Enter an ethics statement for this submission. This statement is required if the study involved:<br><br>• Human participants<br>• Human specimens or tissue<br>• Vertebrate animals or cephalopods<br>• Vertebrate embryos or tissues<br>• Field research<br><br>Write "N/A" if the submission does not require an ethics statement.<br><br>General guidance is provided below. Consult the submission guidelines for detailed instructions. **Make sure that all information entered here is included in the Methods section of the manuscript.**	The museum fossils examined in this study were previously collected by field crews from the Academy of Natural Sciences of Philadelphia and by crews of the Nunavut Paleontological Expedition, the results of which were published in our manuscript's references [25-27]. All necessary permits were obtained for those prior fossil collection campaigns, which complied with all relevant regulations.

Powered by Editorial Manager® and ProduXion Manager® from Aries Systems Corporation

**Format for specific study types**<br><br>**Human Subject Research (involving human participants and/or tissue)**<br>• Give the name of the institutional review board or ethics committee that approved the study<br>• Include the approval number and/or a statement indicating approval of this research<br>• Indicate the form of consent obtained (written/oral) or the reason that consent was not obtained (e.g. the data were analyzed anonymously)<br><br>**Animal Research (involving vertebrate animals, embryos or tissues)**<br>• Provide the name of the Institutional Animal Care and Use Committee (IACUC) or other relevant ethics board that reviewed the study protocol, and indicate whether they approved this research or granted a formal waiver of ethical approval<br>• Include an approval number if one was obtained<br>• If the study involved *non-human primates*, add *additional details* about animal welfare and steps taken to ameliorate suffering<br>• If anesthesia, euthanasia, or any kind of animal sacrifice is part of the study, include briefly which substances and/or methods were applied<br><br>**Field Research**<br><br>Include the following details if this study involves the collection of plant, animal, or other materials from a natural setting:<br>• Field permit number<br>• Name of the institution or relevant body that granted permission	
**Data Availability**<br><br>Authors are required to make all data underlying the findings described fully available, without restriction, and from the time of publication. PLOS allows rare exceptions to address legal and ethical concerns. See the PLOS Data Policy and FAQ for detailed information.	Yes - all data are fully available without restriction

A Data Availability Statement describing where the data can be found is required at submission. Your answers to this question constitute the Data Availability Statement and **will be published in the article**, if accepted.<br><br>**Important:** Stating 'data available on request from the author' is not sufficient. If your data are only available upon request, select 'No' for the first question and explain your exceptional situation in the text box.<br><br>Do the authors confirm that all data underlying the findings described in their manuscript are fully available without restriction?	
**Describe where the data may be found in full sentences. If you are copying our sample text, replace any instances of XXX with the appropriate details.**<br><br>• If the data are **held or will be held in a public repository**, include URLs, accession numbers or DOIs. If this information will only be available after acceptance, indicate this by ticking the box below. For example: *All XXX files are available from the XXX database (accession number(s) XXX, XXX.).*<br>• If the data are all contained **within the manuscript and/or Supporting Information files**, enter the following: *All relevant data are within the manuscript and its Supporting Information files.*<br>• If neither of these applies but you are able to provide **details of access elsewhere**, with or without limitations, please do so. For example:<br><br>*Data cannot be shared publicly because of [XXX]. Data are available from the XXX Institutional Data Access / Ethics Committee (contact via XXX) for researchers who meet the criteria for access to confidential data.*<br><br>*The data underlying the results presented in the study are available from (include the name of the third party*	All relevant data are within the manuscript and its Supporting Information files.

and contact information or URL).<br>• This text is appropriate if the data are owned by a third party and authors do not have permission to share the data.<br><br>* typeset	
Additional data availability information:	

Manuscript
Click here to access/download;Manuscript;PLOSONE formatted Devonian FINAL3.docx

Full Title:

Age is just a number: Examining the preservation of cells and soft tissues in *Bothriolepis* and other Devonian fish

Short Title:

Soft tissue preservation in Devonian fossils

Christopher L. Rogoff[1] and Paul V. Ullmann[2]*

[1] Department of Geology, Rowan University, Glassboro, New Jersey, United States of America

[2] Harold Hamm School of Geology and Geological Engineering, University of North Dakota, Grand Forks, North Dakota, United States America

*Corresponding author

Email: paul.ullmann@und.edu (PVU)

Abstract

Recent microscopy and proteomic studies demonstrate fossil bones can preserve remarkable palimpsests of cellular and soft tissue evolution, yet it remains unclear if there are temporal limits to such preservation. To test this, we demineralized nine bone fragments of *Bothriolepis* and other Late Devonian fish. Removal of apatite from the fossils released numerous microstructures morphologically consistent with vertebrate osteocytes, pieces of blood vessels, and sheets of fibrous bone matrix which were frequently found to exhibit elemental chemistry potentially indicative of partial organic composition. These discoveries extend this style of exceptional preservation to aspidin and dentine and predate all prior reports of similar cellular/soft-tissue preservation in fossil bones by nearly 100 million years, indicating geologic age is not a limiting factor. Thus, this preservation pathway likely began contemporaneously with the evolution of vascularized cellular bone in the early Paleozoic.

Highlights: We report recovery of cellular and soft-tissue microstructures from bones of ~375-million-year-old fish, the oldest such record to date.

Introduction

Though once thought implausible, it is now clear that fossils can preserve exquisite soft tissues not only as mineralized pseudomorphs, but also through retention of endogenous (original) cells, tissues, and their component biomolecules. Recent studies of Mesozoic fossils,

for example, have identified preservation of osteocytes [e.g., 1−3], blood vessels [e.g., 4,5], melanosomes [e.g., 6,7], extracellular fibrous matrix [e.g., 1,8], leukocytes [9,10], and chondrocytes [11,12], several of which have yielded original peptide sequences pertaining to collagen I, myosin, actin, beta tubulin, and other eukaryotic proteins [13−16]. In several cases, independent replication [e.g., 14,16] and use of complementary techniques [e.g., 17,18] have further supported the endogeneity of many recovered microstructures with biochemical and/or geochemical data, requiring paleontologists to revise historical models of fossilization via mere mineral replication [i.e., 19]. Attempts to elucidate soft tissue preservation mechanisms have yielded intriguing clues into biomolecular stabilization reactions [e.g., 20], yet numerous questions remain about how the sources of ancient biomolecules – cellular and soft-tissue microstructures – are able to persist in geologically-ancient fossils [21].

One question of critical importance is if there is a temporal limit to such exceptional preservation or if geologic age bears no influence on this style of exceptional preservation [22]. Following the latter hypothesis, cellular and vascular bone of any geologic age could reasonably be expected to yield endogenous osteocytes, blood vessels, and fibrous matrix.

We tested this hypothesis by performing demineralization assays of 361−378-million-year-old fossil bone specimens, each approximately 100 million years older than any others previously analyzed using this technique (the oldest specimens previously tested are from Permian reptiles [22]). Demineralization assays utilize a weak acid to isolate organic microstructures and oxides lacking divalent cations from biomineralized tissues [21], which can then be analyzed by diverse microstructural, histochemical, immunological, and/or proteomic methods to characterize their morphology, elemental composition, and even molecular composition [e.g., 14,15,23,24].

In this study, ~0.5 cm^3 fragments of nine Late Devonian fish bones and three accompanying sediment samples were demineralized in 0.5 M ethylenediaminetetraacetic acid (EDTA) pH 8.0 (see Materials and methods section below). Recovery of microstructures morphologically consistent with endogenous vertebrate cells (i.e., osteocytes) and/or tissues (i.e., blood vessels) from any of these Devonian fossils would support the conclusion that ancient geologic age does not negatively diminish soft tissue or, by extension, biomolecular preservation potential.

Materials and methods

Materials

The examined fossils include a fragment of dermal (acellular) bone from the heterostracan *Psammosteus*, two pectoral fin spines of the acanthodian *Gyracanthus*, a jaw fragment of the tristichopterid *Hyneria*, scales of the megalichthyid *Megalichthys* and porolepiform *Holoptychius*, and anteroventrolateral plates of the antiarch “placoderm” *Bothriolepis* and an indeterminate bothriolepid (Table 1 and Tables A and B in S1 File). Most of these fossils were recovered from sandy “red bed” fluviodeltaic siltstones at the famous Red Hill locality and other nearby sites in the Catskill Formation in Pennsylvania, but the indeterminate bothriolepid (NUFV 1586) and *Psammosteus* specimens were recovered from sandy fluvial overbank siltstones of the Fram and Nordstrand Point formations, respectively, in Canada [25,26].

Table 1. Summary of cellular and soft tissue recovery from nine Devonian fish bones. Specimens are listed in approximate evolutionary order according to the first appearance of the major clade to which they belong. Abbreviations: ANSP, Academy of Natural Sciences of Drexel University (formerly "of Philadelphia"); NUFV, Nunavut Fossil Vertebrate Collection at the Canadian Museum of Nature in Ottawa, Ontario, Canada; uncat, uncatalogued.

Specimen #	Major Clade	Minor Clade	Taxon	Skeletal Element	Formation	Locality	'Osteocytes'	'Vessels'	'Fibrous Matrix'
NUFV 1587	Pteraspidomorphi	Heterostraci	*Psammosteus* sp.	Dermal bone	Nordstrand Point	Canada, NV2K11	N/A	Rare	Uncommon
ANSP uncat.	"Placodermi"	Antiarchi	*Bothriolepis* sp.	Anteroventro-lateral plate	Catskill	Powys Curve	Abundant	Rare	Uncommon
NUFV 1586	"Placodermi"	Antiarchi	Bothriolepidae indet.	Anteroventro-lateral plate	Fram	Canada, NV0403	Abundant	Rare	Rare
ANSP uncat.	Eugnathostomata	"Acanthodii"	*Gyracanthus sherwoodi*	Pectoral fin spine	Catskill	Red Hill	Abundant	Frequent	Rare
ANSP uncat.	Eugnathostomata	"Acanthodii"	*Gyracanthus sherwoodi*	Pectoral fin spine	Catskill	Red Hill	Abundant	Frequent	Absent
ANSP 23543	Osteichthyes	Porolepiformes	*Holoptychius* sp.	Scale	Catskill	Tioga Rest Stop	Uncommon	Abundant	Rare
ANSP 21165	Osteichthyes	Megalichthyidae	*Megalichthys mullisoni*	Scale	Catskill	Red Hill	Abundant	Uncommon	Rare
ANSP 21165	Osteichthyes	Megalichthyidae	*Megalichthys mullisoni*	Scale	Catskill	Red Hill	Abundant	Uncommon	Rare
ANSP 25034	Osteichthyes	Tristichopteridae	*Hyneria lindae*	Scale	Catskill	Red Hill	Abundant	Uncommon	Rare

The two Canadian Nunavut specimens examined in this study were collected in 2000 (NUFV 1587) and 2004 (NUFV 1586) by Nunavut Paleontological Expedition crews, and the seven specimens from Red Hill and other nearby sites in Pennsylvania were collected between 1994 and 2018 by Academy of Natural Sciences of Philadelphia (ANSP, now "of Drexel University") crews led by Dr. Ted Daeschler. All necessary permits were obtained for those prior fossil collection campaigns, which complied with all relevant regulations [25-27]. For this study, fossil specimens were selected, in part, to achieve a diverse sampling of Devonian "placoderm", stem chondrichthyan, and stem osteichthyan clades (Table A in S1 File). Sediment samples of each entombing sedimentary matrix were also acquired to be tested as negative controls. Fragmented fossil specimens and fossil specimens coated with consolidants (e.g., vinac, butvar,

or similar adhesives) were avoided during specimen selection to preclude glue casts as a potential source of any demineralization products.

Seven of the nine fossil bone samples examined in this study derive from the Famennian (~361 Ma) Catskill Formation (Table 1 and Table A in S1 File). The Catskill Formation primarily consists of grayish-red sandstone, siltstone, and mudstone deposited in prograding deltas [25]. Sediments at each of the three Catskill Formation sites from which the fossils examined herein were collected consist of pale red, very-fine grained sandstone [25]. Indeterminate bothriolepid sample NUFV 1586 was collected from an exposure of the Frasnian (~378 Ma) Fram Formation in Nunavut territory, Canada. The Fram Formation primarily consists of well-indurated sandstones and poorly-indurated siltstones deposited in meandering fluvial channels and adjacent floodplains [26]. Specimen NUFV 1586 was collected from site NV0403, which is one of several Late Devonian sites in the Fram Formation in the southwestern peninsula of Ellesmere Island. Fossiliferous sediments at this locality, and at nearby site NV2K17 from which a representative sediment sample was examined, consist of pale gray-red siltstone interpreted to represent a crevasse splay deposit [26]. The specimen of *Psammosteus* sp. examined in this study was collected from the Frasnian (~375 Ma) Nordstrand Point Formation in Nunavut territory, Canada. The Nordstrand Point Formation consists of green, pale-red, and gray siltstones interbedded with light-gray, very fine to fine sandstones [27]. The entombing sediments at site NV2K11, from which the *Psammosteus* sample was collected, consist of gray siltstone interpreted to have been deposited in a fluvial overbank setting [27].

Methods

Demineralization assays were conducted in a dedicated laminar flow hood within the former Molecular Paleontology Lab at Rowan University, which was dedicated to biomolecular analyses of fossils and in which modern tissue samples were prohibited (this facility has since been repurposed). Incubation in EDTA causes demineralization via chelation of calcium cations from bone, thus leaving behind iron oxides as well as any organic remains not possessing divalent cations [1].

Using a sterilized chisel, small fragments of cortical bone were collected for the demineralization assays. Disposable polypropylene pipettes (1/well) were used for EDTA solution exchanges. Nitrile gloves, a hair net, lab coat, and face mask were worn by all project personnel while conducting the demineralization assays in the Molecular Paleontology Laboratory. EDTA solution was refreshed every 48–72 hours. Most bone samples required ~10 weeks to breakdown into loose "debris", which we collectively refer to in this study as demineralization products (in following of standard practice in this field; e.g., [2]). In contrast, two of the sediment samples (specifically those from the NV2K11 and Red Hill localities) were found to be highly resilient to disaggregation in EDTA, so after 14 weeks they were gradually dissolved over a period of one week in 0.2 M HCl (as in [3]).

For imaging of each sample, the tip was cut off a disposable polypropylene pipette, which was then used to transfer two to three drops (~50 µl) of concentrated demineralization products to standard glass microscope slides. A glass coverslip was then placed over each slide prior to imaging with the Z-stack feature on a Zeiss AxioLab A1 transmitted light microscope with an integrated AxioCam 506 camera. Slides were initially examined under 10X to find larger, potentially-organic remains (i.e., 'vessels'), then 20X and 40X objectives were primarily used to search for and image small potentially-organic remains (i.e., 'osteocytes' and 'fibrous matrix'

fragments). In the absence of biochemical verification of their identities, such purported cells and soft tissue microstructures isolated from the fossils will hereafter be referred to using single quotation marks, in following of common practice in similar prior studies [cf., 8,28,29]. In especially productive samples, a simple grid counting method was used to estimate the approximate number of microstructures present in each slide. This procedure involved tallying the number of each type of microstructure within each field-of-view area under 20X magnification until the entire area of the covered-slipped sample had been searched.

In addition to transmitted light microscopy, demineralization products from select samples were further examined (without gold or carbon coating) using a variable pressure FEI Apreo field-emission gun scanning electron microscope (FEG-SEM) with an Everhart Thornley Detector and an integrated Oxford AZtec energy dispersive x-ray spectroscopy (EDX) probe for elemental analyses. To concentrate and stabilize the isolated demineralization products for imaging by SEM-EDX, they were rinsed three times in Type I (18.2 Ω) water, transferred as above onto ~1 cm^2 silicon chips (as in [8]), and then left to dry for 48 hrs. Structures morphologically consistent with blood vessels required a magnification of 120–1,500X for imaging, whereas those morphologically consistent with osteocytes and fibrous matrix were best viewed around 2,000–6,500X. Select “close up” images were acquired at 10,000–35,000X magnification. SEM images were collected in secondary electron imaging mode at a working distance of ~10 mm, typically using an accelerating voltage of 10 kV; in a few instances, a lower kV setting was used (down to 2 kV).

Results

Examination of demineralization products from the fossil samples revealed abundant microstructures morphologically consistent with vertebrate osteocytes, as well as blood vessels and fibrous extracellular matrix (Fig 1). No similar microstructures were recovered from the three sediment samples, which instead yielded subrounded to angular grains of quartz silt and sand and opaque aggregates of silty mudrock (e.g., Fig A in S1 File). Microstructures recovered from the fossils frequently exhibited various shades of light tan/yellow to dark brown coloration, as in numerous previous studies [e.g., 1,3−5,8,14,23,30]. All examined taxa, regardless of their phylogenetic affinities, yielded identifiable microstructures morphologically consistent with vertebrate cells and soft tissues (Table 1 and Table B in S1 File), supporting the hypothesis that taxonomic identity does not exert a meaningful influence on the extent or style of cellular and soft-tissue preservation [5,21].

Fig 1. 'Osteocytes', 'blood vessel' pieces, and fragments of 'extracellular fibrous matrix' isolated from ~375-million-year-old fish bones and scales. The first two columns display example 'osteocytes', the third shows 'blood vessel' pieces, and the last shows sheets of 'fibrous matrix'. Specimens derive from each taxon by row, as follows: (**A** to **D**) *Bothriolepis*, (**E** to **H**) *Gyracanthus*, (**I** to **L**) *Holoptychius*, (**M** to **P**) *Hyneria*, (**Q** to **T**) *Megalichthys*. Select examples of elongate 'filopodia' off of 'osteocyte cell bodies' are noted by yellow arrowheads in (A) and (N). Note linear fabric of the 'fibrous matrix' fragments, with an example noted between the blue arrowheads in (D). Scale bars as indicated. Fish reconstruction drawings by Nobu Tamura and ABelov2014 (published under CC-BY-SA 3.0 licenses).

The most commonly recovered organic microstructures consisted of a prolate spheroid 'core' surrounded by short to long, thin branching strands. These structures are morphologically consistent with vertebrate osteocytes, or bone cells, which are comprised of a central cell body with emanating filopodia (e.g., fig. 1A in [21]. Most recovered 'osteocytes' were flattened-oblate in shape (*sensu* 30; with a fusiform 'cell body'), though stellate [*sensu* 30] forms (with a spherical 'cell body') were also common, with the central 'cell body' ranging in greatest length from 10–50 µm. 'Filopodia' possessed up to quaternary ramifications (e.g., Fig 1B and 1Q) and were observed to extend from the 'cell body' by up to 120 µm (especially in specimen NUFV 1586). 'Osteocytes' varied widely in color, but most appeared either semitranslucent orange-red, very dark brown, or transparent and colorless, with all three color variants occasionally found within the same sample (in NUFV 1586 and *Hyneria*, similar to the results of [3]).

'Osteocytes' recovered from *Bothriolepis*, the first sample to breakdown, typically retained long, branching 'filopodia', whereas most 'osteocytes' recovered from *Holoptychius* and *Gyracanthus* lacked apparent 'filopodia'. 'Filopodia' were also occasionally encountered as intricate, disarticulated pieces or as entangled bundles in demineralization products from sample NUFV 1586. This specimen also yielded several stellate and flattened-oblate 'osteocytes' with a dark brown central core, which, based on its size and location, appears morphologically consistent with a nucleus (Fig 2B, 2D, and 2E; [cf., 4,30−32]). Several 'osteocytes' recovered from NUFV 1586 and *Hyneria* exhibited sharp transitions from deep red to colorless within the 'cell body' and along 'filopodia' (e.g., Fig 2A and 2C). *Hyneria* and *Megalichthys* yielded the most abundant 'osteocytes' of the taxa examined (i.e., > 200 observed in each slide), most of which retained intricate 'filopodia' (Fig 1M, 1N, 1Q, and 1R). *Holoptychius* yielded the fewest

‘osteocytes’ other than *Psammosteus*, which yielded none. This latter result was expected, as *Psammosteus* possessed acellular bone [33].

Fig 2. ‘Osteocytes’ which display internal heterogeneity, isolated from an anteroventrolateral plate of an indeterminate bothriolepid (NUFV 1586). (**A**) An oblate ‘osteocyte’ partially embedded in a “hazy” fragment of ‘extracellular matrix’. Free-floating, semi-translucent ‘filopodia’ are denoted by white arrowheads, with exemplar transition points between red-orange and semi-translucent colorations denoted by black arrowheads. (**B**) Another oblate ‘osteocyte’ exhibiting a dark central core (red arrow). (**C**) A stellate ‘osteocyte’ exhibiting a semi-translucent region within its ‘cell body’, similar to several ‘filopodia’ of the ‘osteocyte’ shown in (A). (**D** and **E**) Two additional stellate ‘osteocytes’ which possess dark central cores (red arrows), as in the ‘osteocyte’ shown in (B). Scale bars as indicated.

SEM observation revealed ‘osteocytes’ recovered from the bone samples to exhibit a diverse suite of surface textures, ranging from smooth to rough and granular. Crisscrossing linear grooves, potentially representing imprints of collagen fibrils [5,8,34], were commonly seen on the surface of rough-textured ‘osteocytes’ (e.g., Fig 3A and 3B). High magnification (~35,000X) of one exceptionally-preserved *Megalichthys* ‘osteocyte’ revealed the surficial grooves to be recessed into a complex ultrastructure of minute, ~100 nm, blade-like crystallites of iron oxide (see below) oriented at various directions within patches across the ‘cell body’ (Fig 3B). Alternatively, the ‘cell bodies’ of a few ‘osteocytes’ recovered from *Bothriolepis*, *Megalichthys*, and *Hyneria* were coated by larger, ~1–5 µm, plate-shaped crystals of iron oxide (see below).

Fig 3. Scanning electron micrographs of 'osteocytes', 'blood vessel fragments', and fragments of 'fibrous matrix' recovered from Devonian fish scales and bones. (**A** and **C**) Two oblate 'osteocytes' retaining minute 'filopodia' emanating from the 'cell body' (yellow arrowheads), which were recovered, respectively, from a scale of *Megalichthys* and an anteroventral plate of an indeterminate bothriolepid (NUFV 1586). (**B**) Higher magnification of the 'cell body' in (A), revealing crisscrossing grooves (red arrowheads) across its surface. (**D** and **G**) Two cylindrical 'blood vessel' fragments recovered from a fin spine of *Gyracanthus*. (**E** and **H**) Higher magnification images of the 'vessels' shown in (D and G), revealing their alternative hollow (D) and permineralized (G) forms and smooth yet fibrous surface textures. The permineralized vessel cast shown in (G and H) also possesses a sporadic coating of quartz crystals ~1–5 µm in size (pink arrowheads). (**F** and **I**) Two sheets of rough, fibrous, 'extracellular matrix' recovered, respectively, from a scale of *Holoptychius* and an anteroventrolateral plate of an indeterminate bothriolepid (NUFV 1586). The blue arrowheads in (F and I) denote the linear fabric of each 'tissue' fragment. Scale bars as indicated.

Cylindrical microstructures morphologically consistent with blood vessel fragments were found in all nine fossil samples (e.g., Fig 1C, 1G, 1K, 1O, and 1S). These fragments varied in length from ~100–1,400 µm with diameters typically ~20–100 µm. Many 'vessels' appeared hollow with thin walls, though 'vessels' apparently infilled with, or casted by, opaque mineral(s) [cf., 8] were also common. Branching (e.g., Fig 1G and 1O) and anastomosing (e.g., Fig 1K) morphologies were both commonly seen, including up to tertiary ramifications. Hollow, semitranslucent 'vessel' fragments ranged in color from light gray to dark red-brown, with most exhibiting a reddish hue. This style of preservation was especially common in *Bothriolepis* and

Megalichthys (e.g., Fig 1C and 1S). *Holoptychius* and *Gyracanthus* yielded the most abundant 'vessel' fragments, while *Bothriolepis*, NUFV 1586, and *Psammosteus* yielded very few 'vessels' (Table 1). Most 'vessel' fragments recovered from these taxa appeared weakly transparent and dark red. Recovery of 'vessel' fragments but not of 'osteocytes' from the specimen of *Psammosteus*, despite an equally exhaustive search, is consistent with the vascular yet acellular nature of bone in this heterostracan [33]. 'Vessel' pieces recovered from *Bothriolepis* appeared more brittle and fractured than those from the other fossil specimens. All samples which yielded dark 'vessel' casts, namely those of *Gyracanthus*, *Holoptychius*, and *Megalichthys*, also yielded light gray, semitranslucent 'vessels' with a "speckled" appearance (e.g., Fig 1G).

When viewed via SEM, 'vessel' surface textures typically appeared rough, fibrous, and/or microgranular (e.g., Fig 3D and 3E). A minority of 'vessel' fragments, primarily permineralized casts, exhibited a more jagged texture resulting, in part, from a sparse coating of minute, euhedral crystals (e.g., Fig 3H; see below). At high magnification, both hollow and permineralized 'vessel' fragments commonly exhibited a fabric of curvilinear ridges and grooves across their external surfaces (e.g., Fig 3E).

Although fibrous (proteinaceous) matrix represents the most abundant organic microstructure within cortical bone during life, 'matrix' fragments were the least common type of potentially-endogenous microstructure found. 'Fibrous matrix' fragments were identified as mesh-like sheets of semitranslucent 'tissue' (e.g., Fig 1D and 1L) typically ~60–160 μm in greatest dimension (though pieces > 300 μm in length were also occasionally observed). 'Matrix' fragments ranged from essentially colorless (in all taxa except *Megalichthys*) to light to dark yellow (in NUFV 1586) or red-brown (primarily in *Megalichthys*; e.g., Fig 1T). The

Psammosteus sample yielded all three of these color variants. A number of colorless ‘fibrous matrix’ sheets recovered from *Gyracanthus* were partially surrounded by semitranslucent, red-brown, erratically-shaped “patches” of remaining bone mineral (e.g., Fig 1H). A few pieces of ‘matrix’ contained embedded ‘osteocytes’ (primarily in sample NUFV 1586), but this was rarely observed. *Bothriolepis* and *Psammosteus* were found to yield the most ‘matrix’ pieces, yet even these samples each fell within the Uncommon category (Table 1). The *Hyneria* sample yielded only very small fragments of ‘fibrous matrix’.

Under SEM, ‘matrix’ pieces were found to be of random shapes characterized by a uniform lattice structure composed of a linear fabric of rough, parallel-oriented ‘fibers’ (e.g., Fig 3F and 3I). High magnification revealed such fragments to possess minute, pore-like, open spaces between long, anastomosing ‘fibers’.

Energy dispersive x-ray spectroscopy (EDX) analyses revealed many of the isolated cellular and soft-tissue microstructures to possess elemental profiles potentially suggestive of partial organic (i.e., endogenous) composition. This conclusion is primarily based on carbon (C) content, which was frequently found to be > 5 wt.% and occasionally > 10 wt. % (e.g., Fig 4C and 4I). The highest observed weight percentages of C occurred in several ‘vessel’ fragments, which presented C > 20 wt.% (e.g., Fig 4F). It is important to note, however, that these weight percentages for C (and nitrogen [N] and oxygen [O]) should be taken as rough estimates due to the difficulty of acquiring accurate measures of the weight percentages of low atomic number elements via EDX (due to their low photon energy, as well as potential peak shifts and interferences with L-, M-, and N-shell x rays of heavier elements [35]).

Fig 4. Scanning electron microscopy-energy dispersive x-ray spectroscopy (SEM-EDX) analyses of exemplar cellular and soft tissue microstructures recovered from Devonian fish scales and bones. (**A** to **C**) Elemental composition results of EDX on an 'osteocyte' recovered from a scale of *Megalichthys*. (**D** to **F**) Elemental composition results of EDX on a 'blood vessel' fragment recovered from a dentary of *Hyneria*. (**G** to **I**) Elemental composition results of EDX on a shard of 'fibrous matrix' recovered from a scale of *Holoptychius*. Note the general abundance of iron (Fe) and oxygen (O) in each sample, as well as noteworthy weight percentages for carbon (C), potentially suggestive of partial organic composition. Scale bars as indicated. Fish reconstruction drawings by Nobu Tamura and ABelov2014 (published under CC-BY-SA 3.0 licenses).

Generally speaking, all three recovered microstructure types ('osteocytes', 'vessels', and 'fibrous matrix') exhibited similar elemental compositions. Both point analyses (Fig 4) and elemental maps (Figs B-D in S1 File) identified most isolated 'cells' and 'soft tissues' to be primarily composed of iron (Fe), O, C, and silicon (Si), with varying minor amounts of calcium (Ca), N, aluminum (Al), and sodium (Na). As in several previous EDX studies of fossil bone demineralization products [5,8], Fe and O were found to frequently comprise over 50 wt.% of each microstructure, occasionally even over 90% (e.g., several 'osteocytes' recovered from *Megalichthys*). Repetition of our initial 10 kV analyses at higher and lower kV settings (15 kV and 5 kV, respectively) did not drastically alter the weight percentage readouts for Fe. Specifically, these re-analyses found Fe to vary in weight percent by only 1.6−7.5 wt.%, around an average difference of only 2.7 wt.%. Thus, we designate the 10 kV Fe readings to be accurate.

Magnesium (Mg), potassium (K), and phosphorus (P) were generally observed to be present in low weight percentages (Fig 4).

Among the three potentially-endogenous microstructure classes identified, ‘osteocytes’ were found to possess the most variety in elemental composition. Specifically, the ‘cell bodies’ of most ‘osteocytes’ were characterized primarily by high signals for Fe and O (e.g., Fig B in S1 File), indicating the probable presence of goethite [cf., 20,36]. However, a few *Hyneria* ‘osteocytes’ instead yielded high signals for Si and O (e.g., Fig D in S1 File). ‘Osteocytes’ of this latter type exhibited a smoother surface texture and lacked elongate ‘filopodia’, which, in combination with the EDX results, implies that they likely represent quartz casts of cellular lacunae. Where quartz-permineralized casts of osteocyte lacunae have been identified previously, they have been found to possess very short filopodial extensions (e.g., fig. 4B in [37]), most likely due to incomplete permineralization of canaliculi and/or breakage of these extremely thin and brittle projections during the preparation of demineralization products for visualization by transmitted light microscopy and/or SEM.

Most fragments of ‘blood vessels’ and ‘fibrous matrix’ conform to the common trend of high weight percentages for Fe and O described above, but Al, Na, and K were found to be slightly more abundant in ‘vessels’ compared to ‘fibrous matrix’ and ‘osteocytes’. Yet, in every case, each of these elements remained < 16 wt.% (e.g., Fig 4F). Some ‘vessels’ recovered from *Gyracanthus* are coated by a sparse veneer of small (~2–5 μm size), Si-rich crystals of euhedral form consistent with quartz (e.g., Fig 3H). ‘Fibrous matrix’ fragments frequently exhibited a few more weight percent Ca than ‘osteocytes’ and ‘vessels’ (e.g., Fig 4). This trend appears to arise from minor retention of undemineralized bone apatite within the very rough textures of these fibrous ‘tissue’ fragments (Fig C in S1 File).

Discussion

Extending the record

Microstructures that are morphologically consistent with ‘osteocytes’, intricately-branching ‘blood vessels’, and mesh-like fragments of ‘fibrous matrix’ have each previously been recovered from demineralization products of bones from a wide variety of extinct vertebrates, including nonavian dinosaurs, mammals, birds, turtles, fish, and marine reptiles dating as far back as the Permian [21]. Here, we have identified this style of preservation in a diverse suite of Late Devonian fish, none of which had previously been examined by demineralization, namely an acanthodian, heterostracan, antiarch “placoderm”, and several basal osteichthyans. These discoveries extend the temporal record of this type of exceptional preservation of cellular and soft-tissue micromorphology [*sensu* 38] back by approximately 100 million years, supporting the inference [22] that this style of cellular/soft-tissue preservation is independent of geologic age. Our findings also complement those of Ullmann and Schweitzer [21] who found negligible statistical support for preferential preservation of these cellular and soft tissue microstructures across geologic time.

This study also extends the record of this style of cellular and soft-tissue preservation to several bioapatitic tissue types beyond endochondral/perichondral bone, namely osteodentine (in *Gyracanthus*), orthodentine (in *Psammosteus*, *Gyracanthus*, and *Megalichthys*), and aspidin (in *Psammosteus*). Of these three tissue types, only orthodentine had previously been tested for soft-tissue preservation by demineralization, and neither study that previously examined this question

[8,39] recovered any microstructures morphologically consistent with an organic source from teeth (comprised primarily of orthodentine, which lacks osteocytes during life). Consequently, our successful recovery of 'osteocytes', 'vessels', and 'fibrous matrix' from diverse bioapatitic tissue types demonstrates that this style of preservation is also independent of bone/dentine microstructure, which in turn implies that a much larger portion of the vertebrate fossil record may contain informative cellular, soft tissue, and (potentially also) biomolecular data than is currently appreciated.

Inferred preservation pathways

We infer that our successful recovery of intact cellular and soft-tissue microstructures from fossils up to ~378 My in age is likely the product of initial postmortem circumstances which promoted natural fixation and partial mineralization, followed by chemical stability [cf., 2,11,17]. First, the fossils we examined were all collected from sandy, and therefore initially-permeable, fluvial sediments, which have been posited to favor soft tissue preservation as they can facilitate drainage of autolytic enzymes away from a carcass [14]. Moreover, the pale to deep maroon color of the "red bed" sediments entombing all but one of the fossils we examined (that from the Nordstrand Point Formation) typically signifies deposition under semi-arid conditions [40] in conjunction with, or followed by, early-diagenetic oxidation [41], the latter of which is known to facilitate preservation of soft tissues through free-radical-induced [20,36] and oxidative crosslinking (i.e., glycoxidation/lipoxidation) of their component biomolecules [39]. Alongside concurrent precipitation of iron oxides and/or hydroxides (e.g., goethite) onto and within cell membranes [i.e., 8,11,36] and tissue surface layers (i.e., the tunica externa of blood vessels),

these processes can rapidly confer durable, lasting stability to organic microstructures, helping them to persist over geologic timescales [20]. Based on our observations, we infer that all three of these processes likely acted in concert during early diagenesis of the Devonian bones we examined. This conclusion is consistent with both the sedimentary context of the fossils and our EDX findings that many of the recovered microstructures are enriched in iron and oxygen (especially for 'osteocytes' and 'fibrous matrix'; e.g., Fig 4C and 4I).

It is important to note that occasional permineralization of osteocyte lacunae by quartz in the *Hyneria* specimen is not surprising, as alternative forms of preservation of cellular features mere microns away from one another has been encountered previously [e.g., 11]. Further, that the permineralizing phase in this case was quartz indicates that the *Hyneria* specimen experienced greater diagenetic alteration than the others we examined, and that it was exposed to relatively more acidic and silica-rich pore fluids during early diagenesis [cf., 37]. Additionally, the greater abundance of Na, K, and Al in 'vessels' observed via EDX maps and point analyses (e.g., Fig 4D–4F) indicates that these microstructures may have been preserved through partial aluminosilicification [cf., 11] rather than permeation (or casting) with goethite. Cumulatively, then, our results are not only consistent with prior hypotheses about where this type of exceptional preservation may be encountered, but also support the use of the following taphonomic "search criteria" for future studies on soft tissue preservation within fossil bones: burial in sandy/permeable sediments [14], in oxidizing environments [39], and/or in association with abundant iron at the micro-scale [20,36].

Implications for taphonomy and molecular paleontology

Finally, it is important to note how, based on current knowledge, it is clear that 'blood vessels' and 'osteocytes' can potentially be recovered from a diverse array of bioapatitic tissues in fossil bones of any extinct vertebrate which possessed them, specifically since the origin of vascularized bone in pteraspidomorphs in the Early Ordovician [33] and cellular (osteocytic) bone in osteostracans in the early Silurian [42] respectively. Taphonomically, this implies that the early-diagenetic chemical reactions facilitating this type of exceptional preservation described above (i.e., free radical-induced crosslinking [20], sulfurization [43], aluminosilicification [11], and/or glycoxidation/lipoxidation [39]) can operate within most, if not all, bioapatitic tissues, and that they were likely operating as soon as these biomineralized tissues evolved.

These insights have ~~profound~~ implications for the value of the vertebrate fossil record, which paleontologists now recognize to extend to the molecular level. Although it is probable that the average severity of diagenetic alteration generally increases with geologic age [44,45], our findings corroborate those of numerous prior reports [e.g., 1−5,8,14,22,30,32] that have repeatedly found 'osteocytes', 'blood vessels', and 'extracellular matrix' to be remarkably resilient against the long-term rigors of diagenesis, no matter the geologic age of the specimen examined. Because each of these microstructures are, and always were, composed of a rich suite of proteins and other biomolecules during life, many of which also have high preservation potential in their own right (e.g., collagen I, actin, tubulin [15,46], much can potentially be learned from biomolecular analyses of such fossil cells and soft-tissue microstructures. In fact, a growing number of studies have revealed how biochemical (and even proteomic) remnants occasionally identified within fossil 'osteocytes', 'vessels', and similar microstructures can shed light on diverse aspects of the physiology, phylogenetic relationships, and evolutionary history of

extinct vertebrates. For example, collagen I and actin peptide sequences recovered from fossilized ‘extracellular matrix’ and ‘blood vessels’, respectively, have been used to evaluate the phylogenetic relationships of extinct taxa independent of skeletal morphology [13−16]. Similarly, fossil ‘osteocytes’ have been found to be reservoirs of information not only on an individual’s relative age at death [47], but also on bone growth rate [48] and genome size and evolution [e.g., 49,50]. Recognition that ‘osteocytes’ and ‘soft tissues’ can be recovered from fossil bones dating back to the middle Paleozoic implies that each of these types of histologic, paleogenomic, and molecular-phylogenetic analyses could potentially yield informative results from exceptionally-preserved fossils of the earliest vertebrates – an intriguing possibility that would have been considered unfathomable just a few short decades ago.

Acknowledgments

We thank T. Daeschler from the Academy of Natural Sciences of Drexel University for providing the fossil specimen fragments for this study and for granting permissions for destructive analyses, as well as T. Scabarozi, W. Xue, and J. Hettinger from the Rowan University Department of Physics and Astronomy for training and assistance in running the FEG-SEM and EDX analyses.

Author contributions: Conceptualization: PVU; Methodology: CLR, PVU; Investigation: CLR, PVU; Visualization: CLR, PVU; Funding acquisition: PVU; Project administration: PVU; Writing—original draft: CLR, PVU; Writing—review & editing: CLR, PVU.

Data and materials availability: All data are available in the manuscript or the supporting information (S1 File).

References

1. Schweitzer MH, Wittmeyer JL, Horner JR, Toporski JK. Soft-tissue vessels and cellular preservation in *Tyrannosaurus rex*. Science. 2005; 307: 1952–1955.

2. Schroeter ER, Ullmann PV, Zheng W, Schweitzer MH, Lacovara KJ. Soft-tissue, rare earth element, and molecular analyses of *Dreadnoughtus schrani*, an exceptionally complete titanosaur from Argentina. Biology. 2022; 11: 1158.

3. Voegele KK, Boles ZM, Ullmann PV, Schroeter ER, Zheng W, Lacovara KJ. Soft tissue and biomolecular preservation in vertebrate fossils from glauconitic, shallow marine sediments of the Hornerstown Formation, Edelman Fossil Park, New Jersey. Biology. 2022; 11: 1161.

4. Schweitzer MH, Wittmeyer JL, Horner JR. Soft tissue and cellular preservation in vertebrate skeletal elements from the Cretaceous to the present. P R Soc B. 2007; 274: 183–197.

5. Cadena EA. Microscopical and elemental FESEM and Phenom ProX-SEM-EDS analysis of osteocyte- and blood vessel-like microstructures obtained from fossil vertebrates of the Eocene Messel Pit, Germany. PeerJ. 2016; 4: e1618.

6. Lindgren J, Sjövall P, Carney RM, Cincotta A, Uvdal P, Hutcheson SW, et al. Molecular composition and ultrastructure of Jurassic paravian feathers. Sci Rep. 2015; 5: e13520.

7. Pan Y, Zheng W, Moyer AE, O'Connor JK, Wang M, Zheng X, et al. Molecular evidence of keratin and melanosomes in feathers of the Early Cretaceous bird *Eoconfuciusornis*. P Natl Acad Sci USA. 2016; 113: 7900–7907.

8. Ullmann PV, Pandya SH, Nellermoe R. Patterns of soft tissue and cellular preservation in relation to fossil bone tissue structure and overburden depth at the Standing Rock Hadrosaur Site. Cretaceous Res. 2019; 99: 1–13.

9. Plet C, Grice K, Pagès A, Verrall M, Coolen MJL, Ruebsam W, et al. Palaeobiology of red and white blood cell-like structures, collagen and cholesterol in an ichthyosaur bone. Sci Rep. 2017; 7: 13776.

10. Kiseleva D, Shilovsky O, Shagalov E, Ryanskaya A, Chervyakovskaya M, Pankrushina E, et al. Composition and structural features of two Permian parareptile (*Deltavjatia vjatkensis*, Kotelnich Site, Russia) bone fragments and their alteration during fossilisation. Palaeogeogr Palaeocl. 2019; 526: 28−42.

11. Bailleul AM, Zhao Z. Analyses of fossilized chondrocytes in the extinct birds *Yanornis* and *Confuciusornis*: insights on taphonomy and modes of preservation in the Jehol Biota. Front Earth Sci. 2021; 9: 718588.

12. Zheng X, Bailleul AM, Li Z, Wang X, Zhou Z. Nuclear preservation in the cartilage of the Jehol dinosaur *Caudipteryx*. Commun Biol. 2021; 4: 1125.

13. Asara JM, Schweitzer MH, Freimark LM, Phillips M, Cantley LC. Protein sequences from mastodon and *Tyrannosaurus rex* revealed by mass spectrometry. Science. 2007; 316: 280–285.

14. Schweitzer MH, Zheng W, Organ CL, Avci R, Suo Z, Freimark LM, et al. Biomolecular characterization and protein sequences of the Campanian hadrosaur *B. canadensis*. Science. 2009; 324: 626–631.

15. Cleland TP, Schroeter ER, Zamdborg L, Zheng W, Lee JE, Tran JC, et al. Mass spectrometry and antibody-based characterization of blood vessels from *Brachylophosaurus canadensis*. J Proteome Res. 2015; 14: 5252–5260.

16. Schroeter ER, DeHart CJ, Cleland TP, Zheng W, Thomas PM, Kelleher NL, et al. Expansion for the *Brachylophosaurus canadensis* collagen I sequence and additional evidence of the preservation of Cretaceous protein. J Proteome Res. 2017; 16: 920–932.

17. Ullmann PV, Ash RD, Scannella JB. Taphonomic and diagenetic pathways to protein preservation, part II: the case of *Brachylophosaurus canadensis* specimen MOR 2598. Biology. 2022; 11: 1177.

18. Anné J, Canoville A, Edwards NP, Schweitzer MH, Zanno LE. Independent evidence for the preservation of endogenous bone biochemistry in a specimen of *Tyrannosaurus rex*. Biology. 2023; 12: 264.

19. Kolodny Y, Luz B, Sander M, Clemens WA. Dinosaur bones: fossils or pseudomorphs? The pitfalls fo physiology reconstruction from apatitic fossils. Palaeogeogr Palaeocl. 1996; 126: 161–171.

20. Boatman EM, Goodwin MB, Holman H-Y, Fakra S, Zheng W, Gronsky R, et al. Mechanisms of soft tissue and protein preservation in *Tyrannosaurus rex*. Sci Rep. 2019; 9: 15678.

21. Ullmann PV, Schweitzer MH. A statistical meta-analysis of lithologic and other potential controls on fossil bone cellular and soft tissue preservation. PALAIOS. 2023; 38: 246–257.

22. Wiersma K, Läbe S, Sander PM. Organic phase preservation in fossil dinosaur and other tetrapod bone from deep time. In: Gee CT, McCoy VE, Sander PM, editors. Fossilization: Understanding the Material Nature of Ancient Plants and Animals. Baltimore: Johns Hopkins University Press; 2021. pp. 16–54.

23. Surmik D, Boczarowski A, Balin K, Dulski M, Szade J, Kremer B, et al. Spectroscopic studies on organic matter from Triassic reptile bones, Upper Silesia, Poland. PLoS ONE. 2016; 11: e0151143.

24. Fabbri M, Wiemann J, Manucci F, Briggs DEG. Three-dimensional soft tissue preservation revealed in the skin of a non-avian dinosaur. Palaeontology. 2019; 63: 185–193.

25. Daeschler EB, Cressler WL III. Late Devonian paleontology and paleoenvironments at Red Hill and other fossil sites in the Catskill Formation of north-central Pennsylvania. In: Ruffolo RM, Ciampaglio CN, editors. From the Shield to the Sea: Geological Field Trips from the 2011 Joint Meeting of the GSA Northeastern and North-Central Sections. Boulder: Geological Sciety of America; 2011. Field Guide 20.

26. Downs JP, Daeschler EB, Jenkins FA Jr, Shubin NH. A new species of *Laccognathus* (Sarcopterygii, Porolepiformes) from the Late Devonian of Ellesmere Island, Nunavut, Canada. J Vertebr Paleontol. 2011; 31: 981−996.

27. Downs JP, Daeschler EB, Garcia VE, Shubin NH. A new large-bodied species of *Bothriolepis* (Antiarchi) from the Upper Devonian of Ellesmere Island, Nunavut, Canada. J Vertebr Paleontol. 2016; 36: e1221833.

28. Cadena EA. *In situ* SEM/EDS compositional characterization of osteocytes and blood vessels in fossil and extant turtles on untreated bone surfaces; different preservational pathways microns away. PeerJ. 2020; 8: e9833.

29. van der Reest AJ, Currie PJ. Preservation frequency of tissue-like structures in vertebrate remains from the upper Campanian of Alberta: Dinosaur Park Formation. Cretaceous Res. 2020; 109: 104370.

30. Cadena EA, Schweitzer MH. Variation in osteocytes morphology vs bone type in turtle shell and their exceptional preservation from the Jurassic to the present. Bone. 2012; 51: 614–620.

31. Schweitzer MH, Zheng W, Cleland TP, Bern M. 2013. Molecular analyses of dinosaur osteocytes support the presence of endogenous molecules. Bone. 2013; 52: 414–423.

32. Cadena EA, De Gracia C, Combita-Romero DA. An Upper Miocene turtle from panama that preserves osteocytes with potential DNA. J Vertebr Paleontol. 2023; 43: e2254356.

33. Sire J-Y, Donoghue PCJ, Vickaryous MK. Origin and evolution of the integumentary skeleton in non-tetrapod vertebrates. J Anat. 2009; 214: 409−440.

34. Pawlicki R, Nowogrodzka-Zagorska M. Blood vessels and red blood cells preserved in dinosaur bones. Ann Anat. 1998; 180: 73–77.

35. Goldstein JI, Newbury DE, Michael JR, Ritchie NWM, Scott JHJ, Joy DC. Scanning electron microscopy and x-ray microanalysis. 4th ed. New York: Springer Science and Business Media; 2018.

36. Schweitzer MH, Zheng W, Cleland TP, Goodwin MB, Boatman E, Theil E, et al. A role for iron and oxygen chemistry in preserving soft tissues, cells and molecules from deep time. P R Soc B. 2014; 281: 1–10.

37. Boskovic DS, Vidal UL, Nick KE, Esperante R, Brand LR, Wright KR, et al. Structural and protein preservation in fossil whale bones from the Pisco Formation (Middle–Upper Miocene), Peru. PALAIOS. 2021; 36: 155–164.

38. Schweitzer MH. Soft tissue preservation in terrestrial Mesozoic vertebrates. Annu Rev Earth Pl Sc. 2011; 39: 187–216.

39. Wiemann J, Fabbri M, Yang T-R, Stein K, Sander PM, Norell MA, et al. Fossilization transforms vertebrate hard tissue proteins into N-heterocyclic polymers. Nat Commun. 2018; 9: 4741.

40. Guo X, Retallack GJ, Lü B, He L, Wang R, Song H. Paleosols in Devonian red-beds from northwest China and their paleoclimatic characteristics. Sediment Geol. 2019; 379: 16−24.

41. He W, Yang Z, Du H, Hu J, Zhang K, Hou W, et al. Micro-mechanisms and implications of continental red beds. Minerals. 2022; 12: 934.

42. Haridy Y, Osenberg M, Hilger A, Manke I, Davesne D, Witzmann F. Bone metabolism and evolutionary origin of osteocytes: Novel application of FIB-SEM tomography. Sci Adv. 2021; 7: eabb9113.

43. McNamara ME, Orr PJ, Kearns SL, Alcalá L, Anadón P, Peñalver-Mollá E. High-fidelity organic preservation of bone marrow in ca. 10 Ma amphibians. Geology. 2006; 34: 641–644.

44. Hendy J, Welker F, Demarchi B, Speller C, Warinner C, Collins MJ. A guide to ancient protein studies. Nat Ecol & Evol. 2018; 2: 791−799.

45. Slater TS, Edwards NP, Webb SM, Zhang F, McNamara ME. Preservation of corneous β-proteins in Mesozoic feathers. Nat Ecol & Evol. 2023; 7: 1706−1713.

46. Wang S-Y, Cappellini E, Zhang H-Y. Why collagens best survived in fossils? Clues from amino acid thermal stability. Biochem Bioph Res Comm. 2012; 422: 5–7.

47. Padian K, Lamm E-T. Bone Histology of Fossil Tetrapods: Advancing Methods, Analysis, and Interpretation. Berkeley: University of California Press; 2013.

48. Cubo J, Roy NL, Martinez-Maza C, Montes L. Paleohistological estimation of bone growth rate in extinct archosaurs. Paleobiology. 2012; 38: 335−349.

49. Organ CL, Shedlock AM, Meade A, Pagel M, Edwards SV. Origin of avian genome size and structure in non-avian dinosaurs. Nature. 2007; 446: 180−184.

50. Davesne D, Friedman M, Schmitt AD, Fernandez V, Carnevale G, Ahlberg PE, et al. Fossilized cell structures identify an ancient origin for the teleost whole-genome duplication. P Natl Acad Sci USA. 2021; 118: e2101780118.

Supporting information

S1 File. Supplemental details on demineralization assay results, including four supplemental figures (Figs A-D), two supplemental tables (Tables A and B), and supplementary references.

Fig 1

Click here to access/download;Figure;Fig1.tif

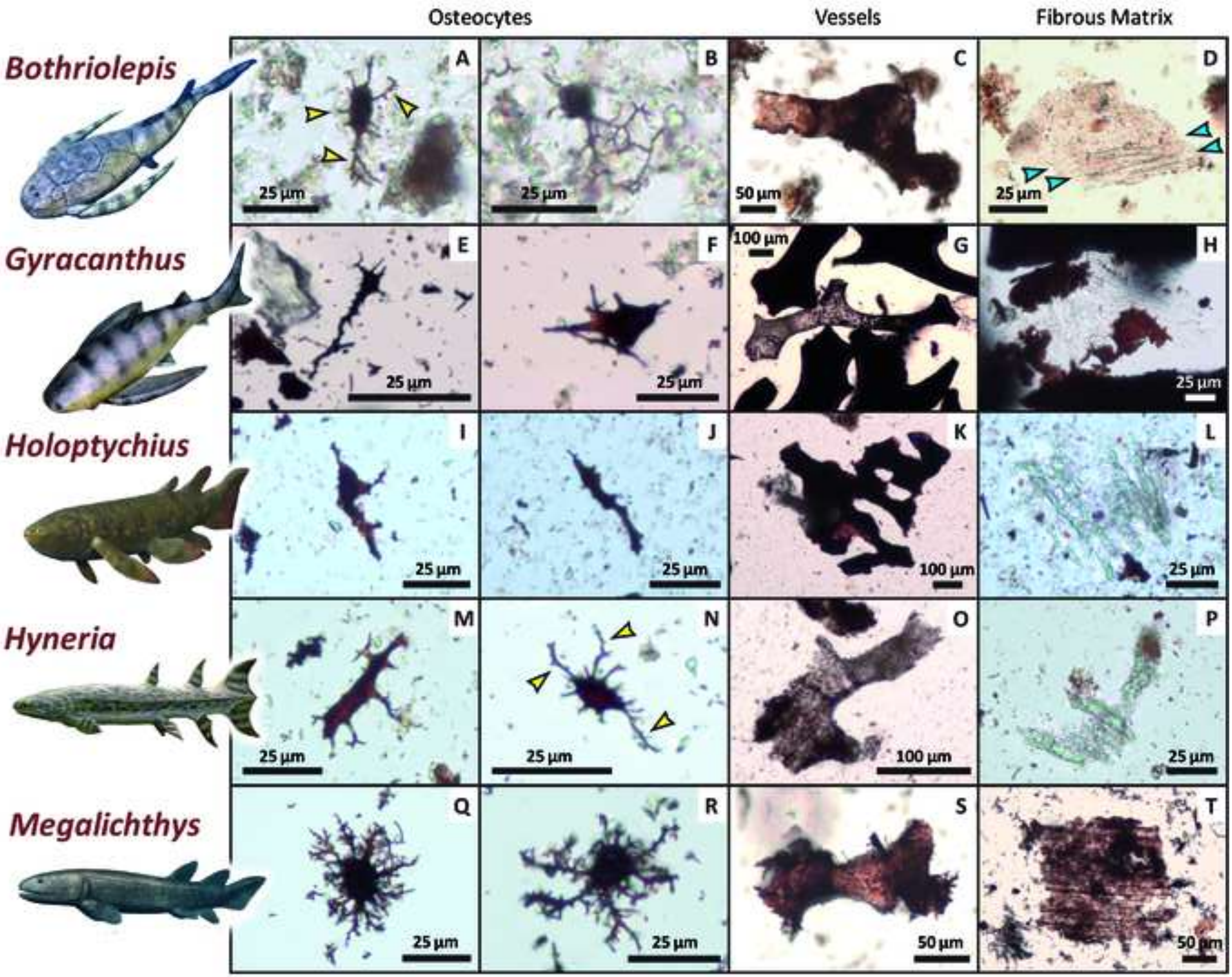

Fig 2

Click here to access/download;Figure;Fig2.tif

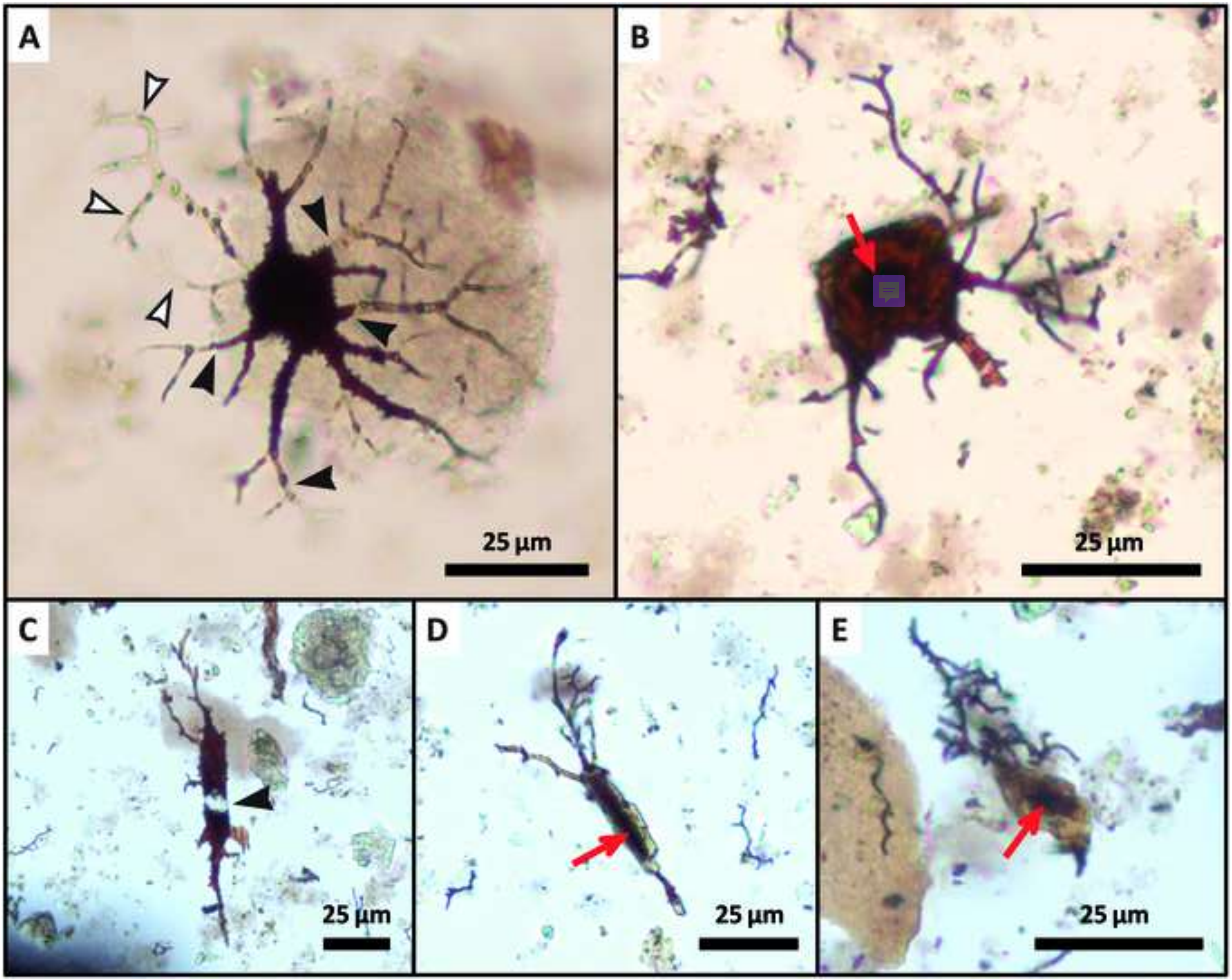

Fig 3

Click here to access/download;Figure;Fig3.tif

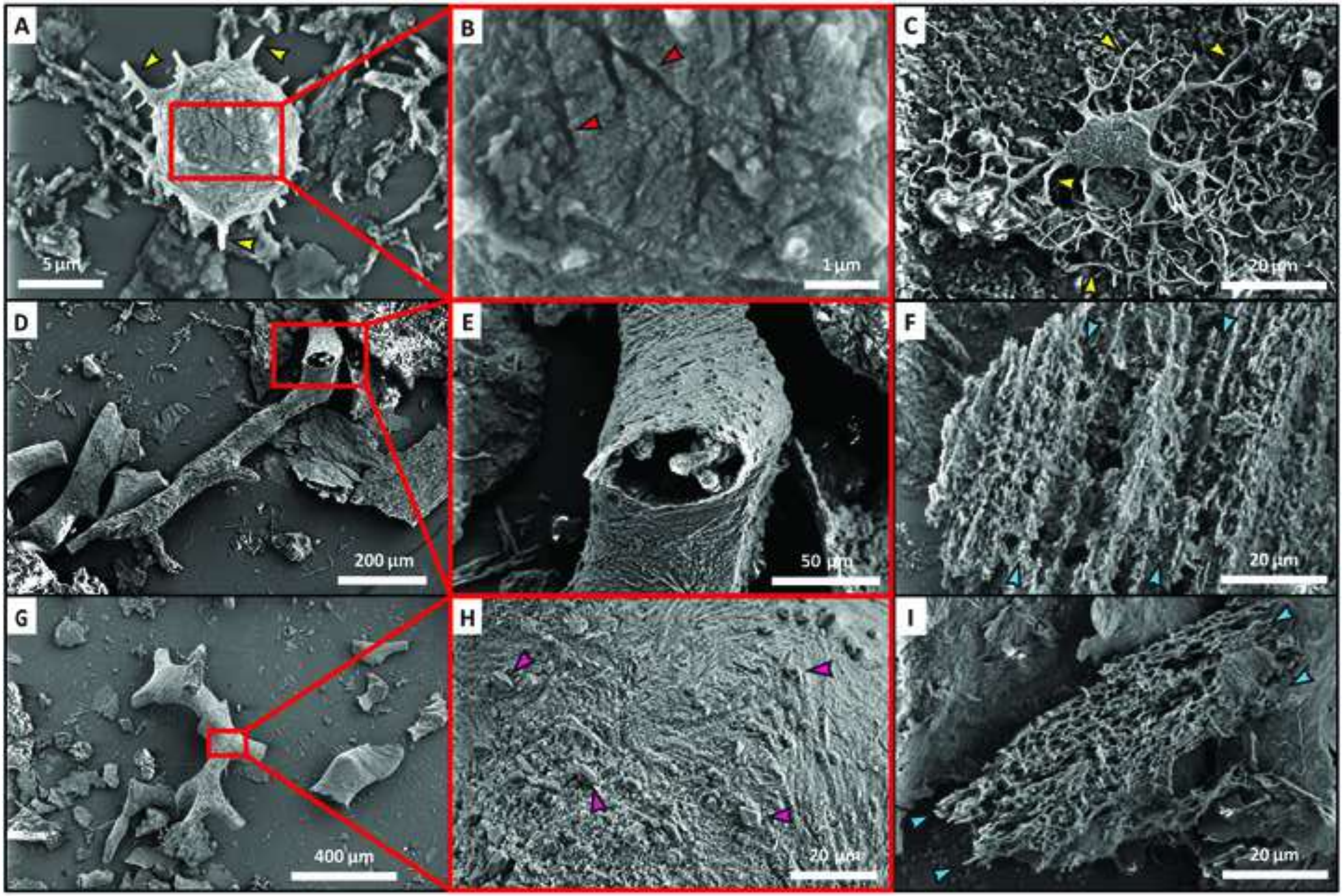

 Click here to access/download;Figure;Fig4.tif

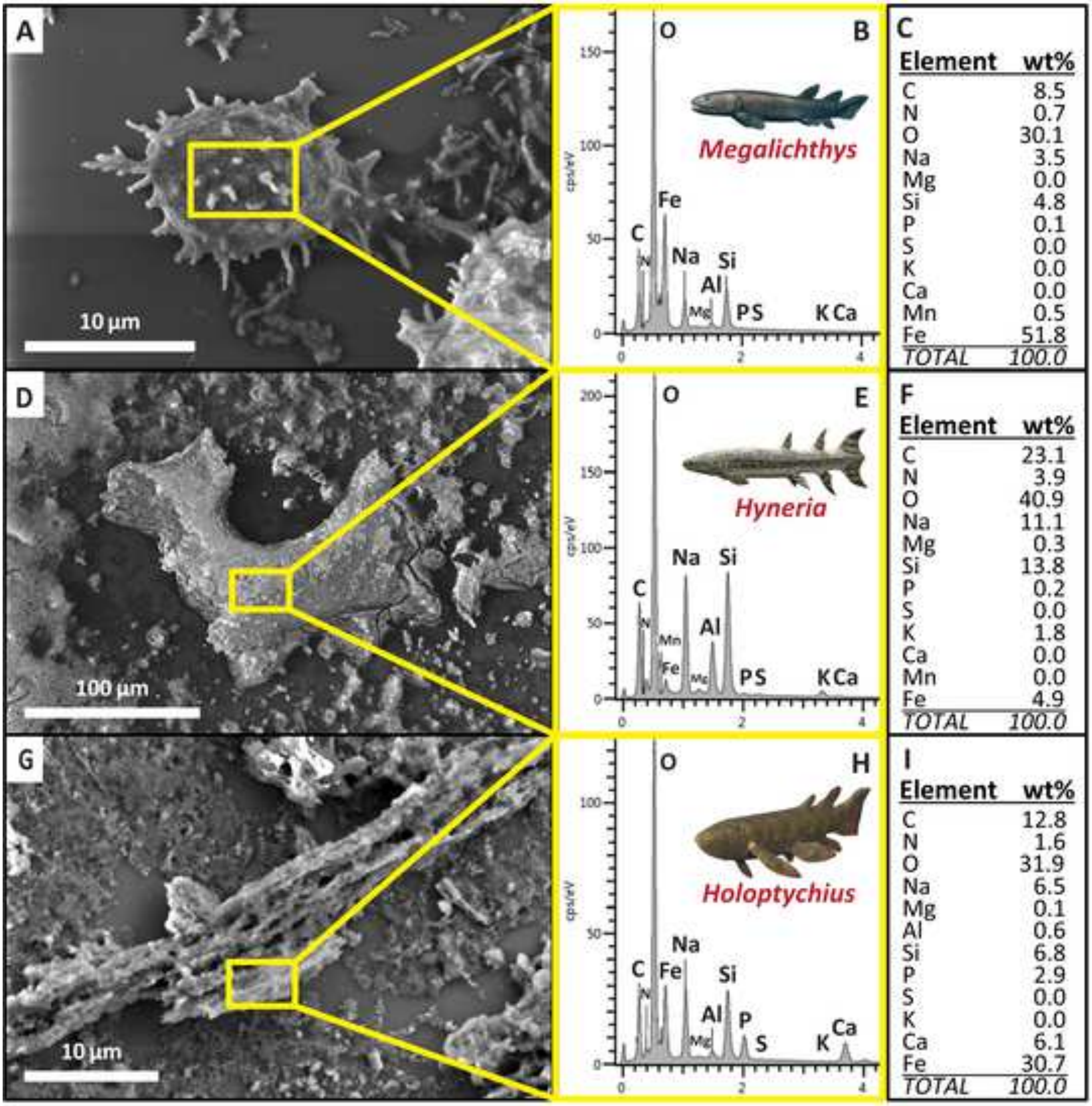

Click here to access/download
Supporting Information
Rogoff&Ullmann_PLoSONE_supplementary_materials_FINAL.docx
