# Supplementary material for: Age is just a number: Examining the preservation of cells and soft tissues in Bothriolepis and other Devonian fish
Source: PLoS One. 2025 Dec 3;20(12):e0335783. doi: 10.1371/journal.pone.0335783 (PMC12674576; doi:10.1371/journal.pone.0335783)
Supplement: Attachment — Submitted filename: Point by Point.docx [file pone.0335783.s004.docx]

**Rogoff and Ullmann Devonian soft tissues manuscript**

**Response to Narrative Comments by the Reviewers**

**COMMENTS BY REVIEWER #1 (CADENA):**

Reviewer #1, Comment #1, Lines 20-21: “well, be specific. Because temporality has been previously tested in different studies, i.e: Cadena & Schweitzer, 2012, Fig. 4. So, be specific for the Paleozoic (Devonian).”

Response: We acknowledge the reviewer’s note that prior studies have also used demineralization assays to investigate the question of how far back cells and soft tissues can be preserved in the fossil record. We could not find a clean, clear way to incorporate the reviewer’s suggestion into the first sentence of the abstract, so we instead inserted a new second sentence into the abstract that qualifies the first sentence by noting how the primary question of our study was about the middle Paleozoic. The abstract thus now begins with these two sentences, as follows: “Recent microscopy and proteomic studies demonstrate fossil bones can preserve remarkable palimpsests of cellular and soft tissue evolution, yet it remains unclear if there are temporal limits to such preservation. For instance, it remains unknown if fossil cells and soft tissues can be recovered from fossil bones of the earliest vertebrates from the middle Paleozoic.”

---

Reviewer #1, Comment #2, Line 22: “are you 100% sure it is just apatite?. Remember that fossilized bone can be mineralized in several other phosphastes (hydroapatatite, francolite). Then, use "phosphates" instead”

Response: Accepted. We have changed “apatite” to “phosphates” in the sentence.

---

Reviewer #1, Comment #3, Lines 50-53: “This is not new. It has been tested and demonstrated for Mesozoic and Cenozoic. Then, the hypothesis should be..."it is the preservation of bone microstructures can be traced and occurs in different groups of Paleozoic vertebrates?"”

Response: We agree with the reviewer that we are not the first researchers to test the question of how far back this style of exceptional preservation goes. Therefore, we have revised the hypothesis stated in the first sentence of the paragraph to focus more clearly on whether these types of microstructures can be recovered from fossil specimens from the middle Paleozoic. The revised sentence now reads as follows: “One question of critical importance is if geologic age bears negligible influence on this style of exceptional preservation, meaning that cells and soft tissue microstructures could be viably recovered from the earliest vertebrates possessing cellular, vascularized bone (i.e., osteostracans in the middle Paleozoic) [22].”

---

Reviewer #1, Comment #4, Line 98: The word “Sediment” is highlighted.

Response: Please see our response to Reviewer #1 Comment #5.

---

Reviewer #1, Comment #5, Line 106: “Sediments of rock matrix? sediments are unconsolidated”

Response: We understand and agree with the reviewer about this distinction, and have changed “sediments” to “rock matrix” or “rocks” throughout the manuscript (including in original draft lines 98, 113, 114, 115, 119, and 173 which were tagged by the reviewer’s use of highlighting and similar comments). Additional similar revisions were made beyond the reviewer’s flagged instances, specifically in revision draft lines 67, 142, and 394, in which we also replaced “sediments” with “rocks” or “rock matrix”. The word “initially” was also added into revision draft line 419 to clarify that we are referring to sediments before they became lithified. The specific sentence marked by this reviewer comment, at original draft line 106, has been entirely rewritten in response to a comment from another reviewer. The rewriting was aimed at providing greater clarity about the number and source of our rock matrix samples. The sentence now reads as follows: “As negative controls, we also acquired and demineralized one rock matrix sample from the fossiliferous horizon at one of the source fossil localities in each of the three geologic formations (Catskill, Fram, Nordstrand Point) studied herein.” We hope that this revised sentence makes it clearer that we tested three rock matrix samples total.

---

Reviewer #1, Comment #6, Line 113: The word “sediments” is highlighted.

Response: Accepted. Please see our response to Reviewer #1 Comment #5.

---

Reviewer #1, Comment #7, Line 114: The words “sediment sample” are highlighted.

Response: Accepted. Please see our response to Reviewer #1 Comment #5.

---

Reviewer #1, Comment #8, Line 115: “then they are rocks.”

Response: Accepted. Please see our response to Reviewer #1 Comment #5.

---

Reviewer #1, Comment #9, Line 119: The word “sediments” is highlighted.

Response: Accepted. Please see our response to Reviewer #1 Comment #5.

---

Reviewer #1, Comment #10, Line 171: “''"”

Response: Quotation marks are not needed in this sentence when we mention the terms osteocytes, blood vessels, and fibrous extracellular matrix, because this sentence is merely stating that the “demineralization products” (the term we used in the beginning of the sentence) that we recovered appear similar to (for-certain, modern) vertebrate osteocytes, blood vessels, and fibrous matrix in extant bone.

---

Reviewer #1, Comment #11, Line 171: “'''”

Response: Please see our response to Reviewer #1 Comment #10.

---

Reviewer #1, Comment #12, Line 172: The word “fibrous” is highlighted.

Response: Please see our response to Reviewer #1 Comment #10.

---

Reviewer #1, Comment #13, Line 173: The word “sediment” is highlighted.

Response: Please see our response to Reviewer #1 Comment #5.

---

Reviewer #1, Comment #14, Line 194: The word “osteocytes” is highlighted.

Response: Please see our response to Reviewer #1 Comment #10.

---

Reviewer #1, Comment #15, Line 194: “other type or bone cells?. It sounds weird. Better write it bone cells (osteocytes)”

Response: Accepted, it now reads as “bone cells (osteocytes)”.

---

Reviewer #1, Comment #16, Line 206: “broken”

Response: Accepted, the line was revised to now read as “intricate, broken pieces” instead of “intricate, disarticulated pieces”.

---

Reviewer #1, Comment #17, Lines 276-278: “references supporting this”

Response: Accepted. We have added two new references, specifically Nielsen-Marsh and Hedges (2000) and Gilbert et al. (2005) which discuss the abundance of fibrous (collagenous) matrix in bone by volume compared to other organic microstructures within bone tissue. These represent our new reference numbers 35 and 36. The later reference numbers have all been adjusted accordingly in both the main text and the Supporting Information file.

---

Reviewer #1, Comment #18, Line 429: Strikethrough of the word “profound”.

Response: Accepted. The subjective adjective “profound” has been removed from the sentence.

---

Reviewer #1, Comment #19: “Are you sure these are "central cores", instead they look just as the base of filopodia from the "ventral" surface of the osteocyte. Usually this create a darker "central" shape., specially in unbroken osteocytes.”

Response: We thank the reviewer for this interesting question, which made us carefully consider our use of nouns for describing these intriguing microstructures. Although we do not find the size of the dark spot within these particular ‘osteocytes’ consistent with the base of a ‘filopodial’ extension (because the dark spot appears too broad in each ‘osteocyte’ to be a ‘filopodial’ base), we agree that we do not have enough information from optical microscopy alone to know that the dark spot/portion/entity is literally within the three-dimensional “core” of each ‘osteocyte’. Therefore, we have changed our description of the dark spot to be at the “center” of each ‘osteocyte’ rather than at its “central core”. We feel the noun “center” works in this instance as it fits the visual 2D observation of seeing the dark spot at the visual center of the ‘cell’, as seen for example in our Figures 2B and 2E. This was the best alternative noun we could think of to describe the visual position of the observed dark spot in these ‘osteocytes’.

---

**COMMENTS BY REVIEWER #2:**

Reviewer #2, Comment #1: “I appreciated the opportunity to review this manuscript. Overall, the scientific approach is sound. I do have some specific comments related to how you are reconciling your methods with conclusions, that I expect can be readily addressed based on (perhaps) subtle rewording and/or the inclusion of a small amount of additional data that you already have.”

Response: We thank the reviewer for taking the time to review our manuscript, and we are happy to address these comments to ensure our data and conclusions are conveyed as precisely as we can make them. Please see our responses to the reviewer’s specific comments below, which explain how we have attempted to increase the clarity of our conclusions and more clearly reconcile them with our methods.

---

Reviewer #2, Comment #2: “You comment on the presence of C in the various tissue remnants, observed by EDS. As you note, the values identified are clearly above background, and I fully accept that there is C in these samples - so this is not a challenge to the conclusion of C being present. However, it may help with your argument to explicitly comment on the fact that EDS typically records several % background C levels due to vacuum greases, etc., evaporated into the system (relevant to lines 300-303). I think acknowledging this and perhaps adding a citation relevant to the system you were using and the relevant environmental settings within the system during your experimental work could help support your case here. (You could also do EDS on a blank sample under the same conditions to test for this.)”

Response: We thank the reviewer for this reminder about other potential sources of inflated and/or stochastically-influenced C signals via EDX, which we were aware of but did not recall at the time of writing to include in our manuscript. Since our manuscript was initially submitted, both of us (CR and PVU) have now moved to other institutions (another university and museum), so although we would definitely enjoy returning to the same SEM-EDX at Rowan University to run a blank sample, it is no longer feasible for us to do so. Thus, we have followed two of the alternative recommendations the reviewer made, specifically (1) to add a note acknowledging this additional influence on C signals during EDX into the text, and (2) to provide a few additional details on the environmental settings of the SEM-EDX while we were performing these analyses. Regarding the former, we added the idea of potential vacuum gas contamination into the end of the sentence at original lines 300-303 marked by the reviewer; the revised sentence now reads as follows: “It is important to note, however, that these weight percentages for C (and nitrogen [N] and oxygen [O]) should be taken as rough estimates due to the difficulty of acquiring accurate measures of the weight percentages of low atomic number elements via EDX (due to their low photon energy, as well as potential peak shifts and interferences with L-, M-, and N-shell x rays of heavier elements [37]) and the potential for minor inflation of C signals stemming from residual organic gas(es) and/or grease within the vacuum chamber of an SEM during analyses [38].” A new reference 38 (Bruker 2011) was added which states this possible source of inflation of C signals during EDX, and all later reference numbers (including in the Supporting Information file) were renumbered accordingly. Regarding the latter, we added a new closing sentence to the end of the Methods section which provides further metadata-sourced settings about the parameters we used while running EDX: “EDX analyses were performed under these operating conditions at an incident angle of 35° to the stage and over reading times ranging from 25–50 s.”

---

Reviewer #2, Comment #3: “Lines 318-320 say that both types of elemental analyses (EDS spots, maps) support the presence of Si in the tissue remnants. As a reader of your draft, all I can see as supporting evidence are the three sets of %s from EDS spot analyses in Fig 4 and the maps in Supp Fig B - and these, in combination, do not support that claim. Based on your descriptions of these tissue remnants and my own work with them, I interpret them to largely be relatively low-density, partially organic, and in some cases, hollow. As a result, many of them contain a relatively small amount of matter for the electron beam to interact with, which is important at high voltages, longer dwell times, and high current densities, such as those with EDS spot collection. Hence, noting that you deposited the samples on Si wafers, and considering what the sample materials are, it wouldn't surprise me to observe Si in the EDS spots, in particular. My concern is that the electron beam might have been interacting with the Si substrate and returning a Si signal in some cases that is not true to the tissue remnants. This would be my interpretation when I compare these figure sets, where the map set in Supp Fig B shows the small amounts of both the O and C spatially correlated with the osteocyte structure of interest, but no Si (lower dwell times, lower electron density, etc). Perhaps you have other map sets that more clearly show the Si for samples from the same specimen and this was simply the less favorable map for me, as your reader, to use for a comparison across your EDS spot and map data to analyze whether your data support your conclusion of Si. Alternatively, perhaps I could suggest seeing if you can replicate the EDS spot findings specific to Si contents by depositing the tissue remnants on C sticky tape instead (in this case, the C values wouldn't be reliable, of course!).”

Response: Although we would normally entertain re-running of the samples via EDS on the same machine with them mounted to sticky tape instead, as mentioned in our response to the previous comment, both of us (CR and PVU) have moved institutions and no longer have feasible access to the same SEM. Moreover, such an analysis would also require demineralizing a new, separate fragment of the fossil(s), as the only demineralization products saved from each sample are those now on the Si chip-mounted SEM stubs analyzed in this study. Thus, this option is unfeasible. We only chose to mention Si because of the occasional observation of higher (~10 to 15) wt.% numbers in select microstructures, primarily in ‘vessels’, not because it makes any organic/biologic sense (we agree with the reviewer that it only makes sense as a diagenetically-sourced element). In surveying all of our elemental maps, it appears that most cases of apparent elevation of Si wt.% readings likely arise from erratic nanocrystalline coatings of ~1-5 micron quartz crystals, like the example shown in Figures 3G-H from a *Gyracanthus* ‘vessel’. These minute quartz crystals were observed to coat both hollow and solid in cross-section ‘vessels’, each of which occasionally gave the slightly higher Si wt.% readings; this combination of findings appears to imply that the apparent elevation of Si signals was minimally influenced by ‘firing through’ hollow vessels to get contamination from the underlying silica chip, which the reviewer expressed as a potential concern. Thus, the minute quartz crystals appear to be a more likely explanation, as best we can tell. To add these collective insights into the manuscript, we removed Si from the list of elements generally composing the recovered microstructures and added a new sentence right afterward discussing these aspects about Si distribution and weight percentages. These two sentences, which span revision draft lines 330-336, read as follows: “Both point analyses (Fig 4) and elemental maps (Figs B-D in S1 File) identified most isolated ‘cells’ and ‘soft tissues’ to be primarily composed of iron (Fe), O, and C, with varying minor amounts of calcium (Ca), N, aluminum (Al), and sodium (Na). A minority of microstructures, most of which were ‘vessels’, were also found to exhibit weight percentages for Si exceeding 10 wt. % (e.g., Fig 4D-F); however, these Si readings appear to likely stem from sparse diagenetic coatings of micron-size quartz crystals (as in Fig 3H, and see below).” The “see below” mentioned at the end of the second sentence refers down to the sentence we had in our original manuscript noting the occasional presence of these quartz micro-crystals: “Some ‘vessels’ recovered from *Gyracanthus* are coated by a sparse veneer of small (~2–5 µm size), Si-rich crystals of euhedral form consistent with quartz (e.g., Fig 3H).” (lines 360-362 in our revised draft).

---

Reviewer #2, Comment #4: “I'm not convinced about the interpretation of the dark central cores in the osteocyte structures as preserved nuclei. Because you have no way to verify with your current methods, perhaps just be sure your language is appropriate (e.g., "possible preserved nuclei"). In a future study, I wonder if FIB might offer an interesting opportunity to section a few of these structures and look for distinct core/shell attributes. Alternatively, there have been some tremendous advances in beamline optics that could possibly allow for spatial analysis, perhaps by nano-FTIR mapping (e.g., LBNL ALS, ANL APS). But on my end, when I look at the images and consider the fact that the structures are round, I feel that I cannot be sure that the central dark core isn't, simply, a region where the round structure is sufficiently thick to obscure light transmission. For example, your arrow in 2D is pointing to a rather long 'nuclear' structure, which seems less probable as a nucleus to me. Alternatively, perhaps in your existing image set you have a sufficient number of photos that you could skim through to see whether you find larger-dimensioned structures from the same specimens that LACK the dark central cores, or even to see whether there is a general trend toward smaller-dimensioned structures lacking cores and larger-dimensioned structures possessing them - these additional lines of consideration with your existing data could, potentially, further support (or refute) your existing conclusion. As it stands, though, my suggestion would simply be to soften the language because your methods have no way of conclusively determining whether these are preserved nuclei.”

Response: We agree that it would be exciting to examine ‘osteocytes’ such as several of the exciting ones shown in our Figure 2 using FIB and/or nano-FTIR in future studies, to more concretely resolve the identity/composition of what, if anything different, is forming the dark ‘spot’ at their visual center. We also agree that we do not have enough information from optical microscopy alone to know that the dark spot/portion/entity is literally within the three-dimensional “core” of each ‘osteocyte’, and believe that our use of the word “core” may have unintentionally been a bit overreaching. Therefore, we have changed our description of the dark spot to be at the “center” of each ‘osteocyte’ rather than at its “central core”. We feel the noun “center” works better as it fits the visual 2D observation of seeing the dark spot at the visual center of the ‘cell’, as seen for example in our Figures 2B and 2E. These changes of “central core” to “center” were also made in the caption to Figure 2. In order to further address the reviewer’s concerns, we also softened the language in the sentence noting the possibility of preserved nuclei by adding the word “potentially” into it, and by taking out the ‘osteocyte’ shown in our Figure 2D from the list of ones with a potential nucleus at the end of the sentence (as we agree that the morphology of the one in Figure 2D differs from those of 2B and 2E and appears less morphologically consistent with a nucleus). That sentence now reads as follows: “This specimen also yielded several stellate and flattened-oblate ‘osteocytes’ with a dark brown center, which, based on its size and location, appears potentially morphologically consistent with a nucleus (Fig 2B and 2E; [cf., 4,30−32]).”

---

Reviewer #2, Comment #5: “This comment is more specific to ensuring your methodology/findings/conclusions supporting your question/hypothesis. You phrase your hypothesis/conclusions a few different ways, and as a result, I admit I am not entirely certain that I am clear on your ultimate conclusion in the context of your question/hypothesis. I believe you are probing whether or not geologic age alone can be a limiting factor in preservation - here, preservation is your word. I'm not sure if you truly mean preservation or perhaps persistence/longevity, which I would think makes more sense in light of your study design. That is, I believe your study design is essentially adding another 100 my to the depth of time from which fossilized soft tissue remnants have been recovered - that those additional 100 my of diagenesis in at least some cases, but maybe many many cases, have little implication on whether we can recover fossilized soft tissue remnants. I absolutely accept that your study design supports this conclusion, and I am excited to see the findings. But I'm not sure that's the same as geologic age "bearing no influence on preservation style" (paraphrased from line 52). Your words, as I read them, indicate you are trying to answer the question as to whether or not geologic age is related to how things preserve and/or fundamentally whether geologic time poses a limit - but I'm struggling to see how sediment types and species types can relate directly to geologic age and preservation style. I absolutely think there's a strong connection between sediment types (and other early post-mortem conditions) and species types (tissue types, physiology, etc.) (all of which you discuss), and I recognize that some of the earliest works in this area indicated upper limits on protein/soft tissue persistence, which have been 'violated' time and again by studies like yours and mine. But I can't see that one additional report extending the recovery of soft tissue remnants back by 100 my would also be enough to say geologic age has no bearing - afterall, vertebrates existed even before the age of the specimens which your team sampled. Fundamentally, I want to be clear I'm not challenging your findings or conclusions - just the presentation of them and how they're tied into your initial 'hypothesis' or research question (and maybe simply rephrasing as a question instead of hypothesis would be best). Would it perhaps be better to rephrase as EITHER simply "these findings further extend the records of persistence deeper into geologic time and suggest that if a limit exists, it has not yet been found" OR "these findings suggest other factors than geologic time may be more relevant to whether fossilized soft tissue remnants can be recovered from extinct life forms" (e.g., tissue structure and composition, physiology). Perhaps even more simply, I'm not sure you can say "geologic age is not a limiting factor" based on this report extending back known records by another 100 my. The only thing needed to resolve this comment would be rewording of relevant sections, and a bit more specificity in the question you are trying to answer (or can answer).”

Response: We thank the reviewer for noting these inconsistencies in our phrasing of our research question and primary conclusion. The reviewer has interpreted our intended ‘hypothesis’ correctly in that we sought to test whether cellular/soft tissue microstructures could be recovered from samples this much older than any others that have been tested via demineralization thus far. As the reviewer also correctly interprets, we did not intend to test whether increasing geologic age leads to a different preservation style(s) – that was not our ‘hypothesis’, and our choice of methods would indeed be insufficient to answer that question. We acknowledge that a few of our ‘conclusion’-style statements regarding geologic age were phrased a bit strongly given that they are based off solely a single study on nine fossil specimens. Therefore, we have tried to (1) soften some of the stronger phrasings throughout the manuscript, such as changing “no influence” to “negligible influence” and new alternative phrasings like “geologic age is a poor predictor...”, and (2) clarify our ‘hypothesis’ and the conclusions we draw from our results. The specific changes we made are listed below by revision draft line numbers:

- Lines 29-31 in the Abstract were softened to say: “These discoveries extend this style of exceptional preservation to aspidin and dentine and predate all prior reports of similar cellular/soft-tissue preservation in fossil bones by nearly 100 million years, indicating that the geologic age of a fossil specimen is a poor predictor of whether or not it will retain potentially-endogenous microstructures.”
- Lines 53-57 stated our ‘hypothesis’, which was almost entirely rewritten to improve its clarity and specificity: “One question of critical importance is if geologic age bears negligible influence on this style of exceptional preservation, meaning that cells and soft tissue microstructures could be viably recovered from the earliest vertebrates possessing cellular, vascularized bone (i.e., osteostracans in the middle Paleozoic) [22].”
- Lines 71-74 included a small revision (of adding the word “significantly”): “Recovery of microstructures morphologically consistent with endogenous vertebrate cells (i.e., osteocytes) and/or soft tissues (i.e., blood vessels) from any of these Devonian fossils would support the conclusion that ancient geologic age does not significantly diminish soft tissue or, by extension, biomolecular preservation potential.”
- Lines 375-378 included a change of “is” to “may be” to soften the language: “These discoveries extend the temporal record of this type of exceptional preservation of cellular and soft-tissue micromorphology [*sensu* 41] back by approximately 100 million years, supporting the inference [22] that this style of cellular/soft-tissue preservation may be independent of geologic age.”
- Lines 448-449 had the phrase “no matter the geologic age of the specimen examined” removed so as to avoid the use of strong wording implying our study fully answers the question of a temporal ‘limit’.

---

Reviewer #2, Comment #6: “On line 99 you say "each entombing matrix", but you only analyzed three matrix specimens. To respond to this comment, I am only looking for better clarity in the writing in the manuscript. While I do think analysis of the actual entombing matrix (not simply matrix from the same area near where the specimens were recovered) would be ideal, I'm not clear from your writing whether this is in fact how you would describe the matrix specimens or not. Further, when I read "each", I expect to see a matrix specimen for "each specimen", but the numbers don't seem to line up to me. Could you please add some additional language to the few locations that discuss the matrix specimens and/or remove language that is too specific and not accurate to ensure that the text is as accurate as possible so that your reader most clearly knows how to interpret your findings?”

Response: We thank the reviewer for pointing out this inconsistency and lack of clarity in our description of the rock matrix samples we examined. We did indeed test three rock matrix samples total, rather than one per fossil specimen. To clarify why we tested three: unfortunately, rock matrix samples were not available from all five localities from which we examined fossil specimens. The fossil specimens were fully prepared prior to sampling as they derived from museum collections, which typically discard associated rock/sediments during the process of fossil preparation. We tried to address this drawback by testing as many associated rock matrix samples as possible, but this still limited us to just one from one of the localities studied in each Formation. Luckily, the three Catskill Formation localities each were noted in prior publications to exhibit essentially the same sedimentology, so our representative rock matrix sample from Red Hill was deemed to be sufficient. The other two formations, Fram and Nordstrand Point, each align “one-to-one" in sampling intensity between fossils and associated rock matrix (1 fossil to 1 rock matrix sample). To clarify these details, we have expanded the details provided in revision draft lines 102-106 and 143-146, the two primary locations in the manuscript that discuss our rock matrix samples. The sentence at line 102 was completely rewritten, and now reads as follows: “As negative controls, we also acquired and demineralized one rock matrix sample from the fossiliferous horizon at one of the source fossil localities in each of the three geologic formations (Catskill, Fram, Nordstrand Point) studied herein.” At line 143, we added more detail, in the form of “three representative”, so that it now reads as follows: “In contrast, two of the three representative rock matrix samples (specifically those from the NV2K11 and Red Hill localities) were found to be highly resilient to disaggregation in EDTA, so after 14 weeks they were gradually dissolved over a period of one week in 0.2 M HCl (as in [3]).”

---

Reviewer #2, Comment #7: “Line 151 - You mention tallying the remnants using a grid overlay, but you have no statistical analyses or counts, etc. Is this perhaps not worth including at all? When I read it, I expected to see the results from the overlay come up somewhere, and they did not.”

Response: We believe our use of the word “grid” in the sentence may have misled the reviewer, as we did not utilize a grid overlay, slide-grid, or well slide with a microgrid for the counting procedure being described. Rather, we simply split the area of the coverslip up into 20X field of view ROIs and counted the number of osteocytes within each ROI. We agree that the use of a grid overlay or slide-grid would be a much more accurate and reproducible means of performing this type of task, so we will look into attempting such techniques in our future demineralization studies. To remove any confusion about the methodology we employed, we removed the word “grid” from the sentence marked by the reviewer, which was the only sentence in the entire manuscript (and Supplemental Information file) that used this term to refer to our method. Removal of this word now clarifies, we believe, that our technique was merely a simple form of a counting procedure. The revised sentence, and the sentence following it which explains the method we employed in greater detail, now read as follows: “In especially productive samples, a simple counting method was used to estimate the approximate number of microstructures present in each slide. This procedure involved tallying the number of each type of microstructure within each field-of-view area under 20X magnification until the entire area of the covered-slipped sample had been searched.”

---

Reviewer #2, Comment #8: “You have a missing closing parenthesis”

Response: Thank you. We added it to revision draft line 200.

---

Reviewer #2, Comment #9: “I have always been curious about the fibers that appear WITHIN the vessel structures - which you can see in your image Fig 3E. I routinely saw these in different specimens when I worked with fossil vessels, and based on my knowledge of vessel tissues, I was never entirely confident in identifying them. However, seeing them consistently revealed in additional studies, like yours, makes me think they truly are preserved endogenous fibers - elastin in the media? collagen in the adventitia? Curious!”

Response: We had not noticed the commonality of such apparent ‘fibers’ among studies, but now paying closer attention to them we agree with the reviewer that they are indeed quite fascinating. The reviewer’s hypotheses about their composition(s) (i.e., elastin or collagen) are intriguing, and we agree it would be exciting to test them via immunological assays, mass spectrometry, and/or other methods in future studies of ‘blood vessels’ recovered from either these or other fossil specimens. Since no specific request was made to revise anything in the text, we have made no changes to the manuscript in response to this comment.

---
